# Role of SUMOylation in differential ERα transcriptional repression by tamoxifen and fulvestrant in breast cancer cells

**DOI:** 10.1038/s41388-018-0468-9

**Published:** 2018-09-06

**Authors:** Tatiana Traboulsi, Mohamed El Ezzy, Vanessa Dumeaux, Eric Audemard, Sylvie Mader

**Affiliations:** 1 Institute for Research in Immunology and Cancer, Montréal, QC H3C 3J7 Canada; 2 Department of Biochemistry and Molecular Medicine, Université de Montréal, Montréal, QC H3C 3J7 Canada; 3 PERFORM Centre, Concordia University, Montréal, QC H4B 1R6 Canada

**Keywords:** Targeted therapies, Breast cancer, Sumoylation

## Abstract

Antiestrogens (AEs) are widely used for treatment of estrogen receptor alpha (ERα)-positive breast cancer, but display variable degrees of partial agonism in estrogen target tissues and breast cancer (BC) cells. The fact that BC cells resistant to selective ER modulators (SERMs) like tamoxifen (Tam) can still be sensitive to pure AEs, also called selective ER downregulators, suggests different mechanisms of action, some of which may contribute to the more complete suppression of estrogen target genes by pure AEs. We report herein that pure AEs such as fulvestrant induce transient binding of ERα to DNA, followed by rapid release after 30–40 min without loss of nuclear localization. Loss of DNA binding preceded receptor degradation and was not prevented by proteasome inhibition. Chromatin was less accessible in the presence of fulvestrant than with estradiol or Tam as early as 20 min following treatment, suggesting that chromatin remodeling by pure AEs at ERα target regions prevents transcription in spite of receptor binding. SUMO2/3 marks were detected on chromatin at the peak of ERα binding in cells treated with pure AEs, but not SERMs. Furthermore, decreasing SUMOylation by overexpressing the deSUMOylase SENP1 significantly delayed receptor release from DNA and de-repressed expression of estrogen target genes in the presence of fulvestrant, both in ERα-expressing MCF-7 cells and in transiently transfected ER-negative SK-BR-3 cells. Finally, mutation V534E, identified in a breast metastasis resistant to hormonal therapies, prevented ERα modification and resulted in increased transcriptional activity of estrogen target genes in the presence of fulvestrant in SK-BR-3 cells. Together, our results establish a role for SUMOylation in achieving a more complete transcriptional shut-off of estrogen target genes by pure AEs vs. SERMs in BC cells.

## Introduction

About 70% of breast tumors are classified as positive for the estrogen receptor alpha (ERα), a ligand-dependent transcription factor that controls expression of proliferative genes in breast cancer (BC) cells [1]. When bound by estrogens, ERα rapidly binds DNA at estrogen response elements (EREs) and recruits cofactors with histone-modifying activities, chromatin remodeling complexes, and the basal transcriptional machinery, resulting in altered expression of target genes [1–3]. Antiestrogens (AEs) are small synthetic molecules designed to compete with estrogens and block ERα transcriptional activity [4–7]. Selective ER modulators (SERMs) like Tamoxifen (Tam) induce gene- and cell type-specific patterns of cofactor recruitment to ERα, leading to estrogenic effects in tissues such as bone and uterus [8–11]. In contrast, fulvestrant was originally described as a “pure” AE as it is antagonistic in these tissues [12, 13]; it is also more efficient than SERMs in suppressing ERα transcriptional activity in BC cells [14, 15]. The observation that BC cells resistant to SERMs can still be sensitive to pure AEs in experimental models [16–18] or in the clinic [19, 20] implies different mechanisms of action, some of which may contribute to the increased transcriptional inhibition by pure AEs vs. SERMs.

Pure AEs are also currently called selective ER downregulators/degraders (SERDs), as they lead to increased turnover of ERα via the ubiquitin—proteasome pathway [6, 7]. ERα degradation likely contributes to the antiestrogenic profile of pure AEs, but ERα levels are significantly depleted only after several hours [21–24], whereas estradiol (E2) or Tam can activate transcription within 1 h [8, 9, 25–27]. After 1 h of fulvestrant treatment in MCF-7 cells, ERα binds to ~ 33% of the regulatory regions bound in the presence of E2 [27], suggesting that transcription of the corresponding genes is prevented via means other than ERα degradation. In addition, pure AEs remain more efficacious than SERMs at suppressing transcription of estrogen reporter genes in HepG2 cells, a model in which they do not accelerate ERα turnover [23, 24].

We previously reported that pure AEs can be distinguished from SERMs by their capacity to induce rapid modification of ERα by SUMO1/2/3 in receptor-positive BC cell lines and in transfected ER-negative cell lines following pure AE treatment [24]. Reporter assays in HepG2 cells showed that abrogating SUMOylation by overexpression of a deSUMOylase partially de-repressed ERα transcriptional activity in the presence of pure AEs [24]. However, the impact of pure AE-induced SUMOylation on transcriptional repression of ERα in BC cells remains uncharacterized, and the mechanisms by which SUMOylation may contribute to the differential properties of pure AEs vs. SERMs are currently unclear.

Herein, we investigated the impact of SUMOylation on the kinetics of ERα association with DNA, on chromatin accessibility and on transcriptional suppression, to better understand the role of this modification in the more complete repression of ERα-mediated transcription in the presence of pure AEs compared with SERMs in BC cells. Our results demonstrate that induction of SUMOylation by pure AEs contributes to their stronger antiestrogenicity compared with SERMs, and that a naturally occurring mutation that abrogates ERα SUMOylation leads to increased transcription of estrogen target genes in the presence of fulvestrant in BC cells.

## Results

### Fulvestrant (ICI182,780) and 4-hydroxytamoxifen exhibit differential regulation of estrogen target genes in MCF-7 cells

**Fig. 1:**
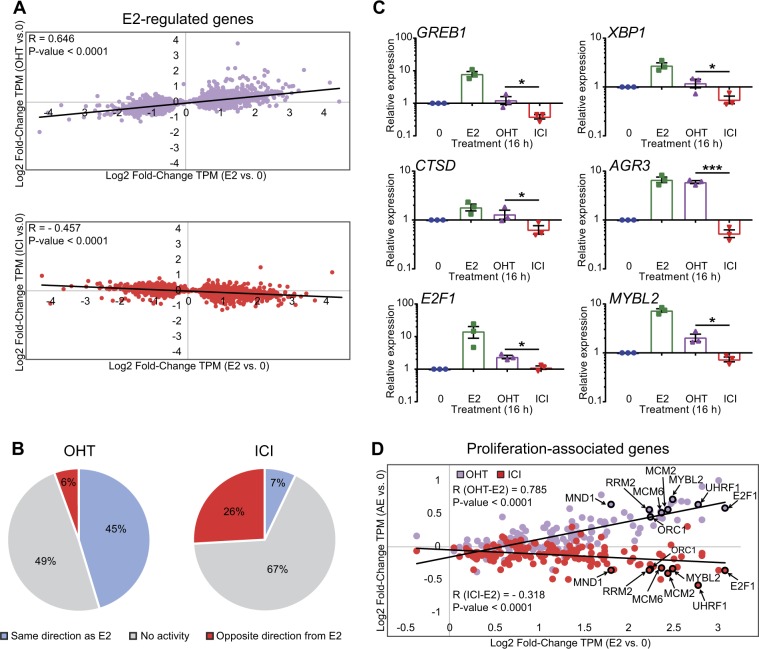
ICI182,780 and OHT exhibit differential regulation of estrogen target genes in MCF-7 cells. **a** RNA-sequencing was performed on MCF-7 cells cultured in estrogen-depleted media and treated for 16 h with estradiol (E2, 5 nM), 4-hydroxytamoxifen (OHT, 100 nM), ICI182,780 (ICI, 100 nM), or vehicle only. Data from three biological replicates were analyzed by Kallisto/Sleuth [28, 29]. Scatter plots of the differential expression values (Log2 fold-change TPM, AE vs. 0) of E2-regulated genes (*q* value < 0.05) by OHT (top) or ICI (bottom) vs. that of E2 are shown. *P* values from an *F* test are indicated. **b** Regulation of E2 target genes by OHT and ICI is shown as pie charts. “Same direction as E2” group: Log2 fold-change TPM (AE vs. 0)/Log2 fold-change TPM (E2 vs. 0) > 20%. “No activity” group: − 20% ≤ Log2 fold-change TPM (AE vs. 0)/Log2 fold-change TPM (E2 vs. 0) ≤ 20%. “Opposite direction from E2” group: Log2 fold-change TPM (AE vs. 0)/Log2 fold-change TPM (E2 vs. 0) < − 20%. **c** Bar graphs showing the gene expression profiles of selected E2 target genes differentially regulated by OHT and ICI (see full list in Suppl. Table 1). mRNA levels from MCF-7 cells treated as in **a** were determined by RT-qPCR. Data points from three independent experiments, as well as means ± SEM, are represented. Asterisks denote significance (one-tailed *t* test): * *P* value < 0.05; *** *P* value < 0.0005. **d** Scatter plot of the differential expression values (Log2 fold-change TPM from the RNA-Seq analysis from **a)** of proliferation-associated genes upon treatment with OHT (purple) or ICI (red) vs. E2. Genes significantly differentially regulated (*q* < 0.05) by OHT and ICI are bolded and labeled. *P* values from an *F* test are indicated

Transcription of estrogen target genes was previously shown by microarray analyses to be repressed more efficiently by the pure AE fulvestrant (ICI182,780) than by various SERMs in the ERα-positive MCF-7 BC cell line [14, 15]. Here, we have used RNA-sequencing to identify estrogen target genes differentially regulated by ICI182,780 and the active Tam metabolite, 4-hydroxytamoxifen (OHT), in MCF-7 cells following 16 h of treatment in estrogen-depleted medium. Analysis of transcriptomes from three independent experiments with Kallisto/Sleuth [28, 29] revealed that E2 regulated a large number of genes (2039 induced and 1878 repressed genes) in a significant manner (*q* < 0.05) compared with non-treated controls. Consistent with previous studies performed at different time points [14, 15], a smaller number of genes (82) were significantly regulated by OHT using the same statistical cutoff, whereas genes significantly regulated by ICI182,780 were rare (12). Using Log2_Fold Change of RNA levels (TPM) in treated vs. vehicle samples as a measure of gene regulation, we observed a positive correlation between regulations by OHT and E2 in a linear regression analysis (*R* = 0.646; *P* value < 0.0001), albeit with much weaker overall regulation by OHT than by E2 (Fig. 1a, top). Conversely, there was an anti-correlation between gene regulations by ICI182,780 and by E2 (*R* = −0.457; *P* value < 0.0001) (Fig. 1a, bottom). Overall, most E2 target genes regulated by OHT were affected in the same direction as E2, and far fewer in the opposite direction (45 and 6%, respectively, Fig. 1b), whereas this proportion was reversed for ICI182,780 (7 and 26%, respectively, Fig. 1b). In addition, 178 genes were differentially regulated by OHT and ICI182,780 (*q* < 0.05, Suppl. Table 1). This included well-characterized direct E2 target genes such as *GREB1*, *XBP1, CTSD*, and *AGR3*, as well as proliferation-associated genes [30] like *E2F1* and *MYBL2* (Fig. 1c,d). Consistent with these observations, MCF-7 cells grew more slowly in the presence of ICI182,780 than OHT (100 nM in estrogen-depleted medium) (Suppl. Figure 1). These results are compatible with previous reports that ICI182,780 blocks MCF-7 cell proliferation with increased efficacy compared with OHT [12], and with the existence of different mechanisms of transcriptional regulation by the two AEs.

### ERα binding to DNA is biphasic in the presence of ICI182,780

**Fig. 2:**
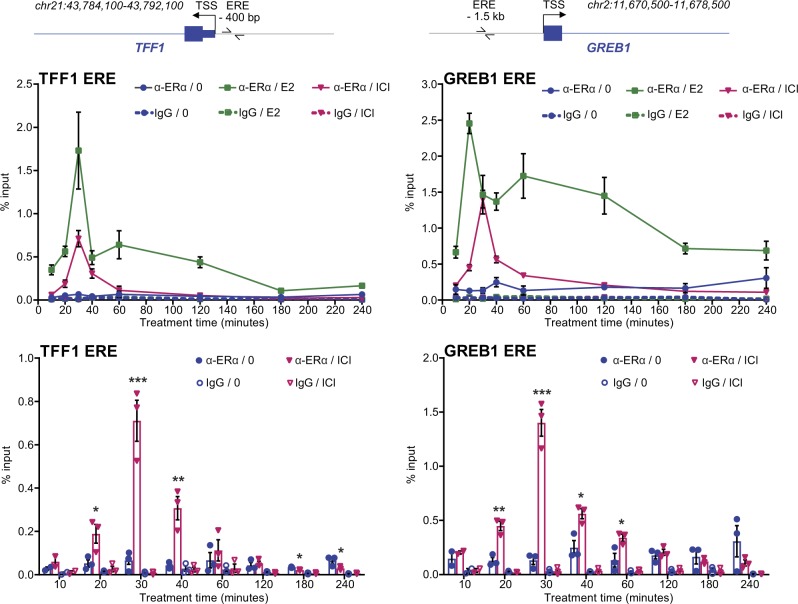
ICI182,780 induces transient binding of ERα to DNA. ERα binding to target gene regulatory regions was determined by chromatin immunoprecipitation (ChIP) in MCF-7 cells treated with estradiol (E2, 5 nM), ICI182,780 (ICI, 100 nM), or vehicle only (0) for the indicated time points. The ERE position relative to the gene TSS is shown. Graphs show the evolution of ERα binding as a function of time for vehicle (blue), E2 (green), and ICI (magenta) conditions, whereas the bar graphs compare ERα binding in the presence of ICI and vehicle. Data points from three independent experiments, as well as means ± SEM, are represented, along with asterisks denoting significance (one-tailed *t* test, ICI vs. 0): * *P* value < 0.05; ** *P* value < 0.005; *** *P* value < 0.0005

Although SERMs and E2 induce binding of ERα to EREs at target gene promoters or enhancers [9, 27], pure AEs were reported to affect binding to DNA to varying extents in different studies. ERα association to DNA was not detected at the *TFF1/pS2* promoter after 3 h of treatment with ICI182,780 by chromatin immunoprecipitation (ChIP) in MCF-7 cells [26], but binding of ERα at this site and at other EREs was observed after 1 h of ICI182,780 treatment in the same cell line [27]. Here, we performed time course ChIP experiments in MCF-7 cells to monitor receptor recruitment at EREs upstream of the E2 target genes *TFF1*, *GREB1*, and *CTSD* in the presence of E2 or ICI182,780 (Fig. 2 and Suppl. Figure 2A). Receptor binding was induced by ICI182,780, with a peak after 30 min of treatment, following kinetics similar to those in the presence of E2. However, binding to DNA in the presence of ICI182,780 was then lost rapidly between 30 and 60 min, with a gradual return to basal levels (or lower) over the next 3 h (Fig. 2 and Suppl. Figure 2A, magenta vs. blue). Neither E2 nor ICI182,780 induced receptor recruitment at control regions located in the *GREB1* and *CTSD* gene bodies (Suppl. Figure 2B). These results reconcile previous observations [26, 27] and contrast the impact of ICI182,780 treatment on ERα association to DNA with that of E2. Indeed, increased ERα binding was observed at all time points with E2, although levels of bound receptor varied over time (Fig. 2 and Suppl. Figure 2A, green vs. blue). These observations, obtained using quantitative real-time PCR, are consistent with the previously described strong recruitment of ERα to the *TFF1* and *CTSD* promoters after 30 min of E2 treatment, followed by decreased association at 1 h observed by semi-quantitative agarose-gel based PCR analysis [8]. However, we did not observe clear cyclical patterns of ERα recruitment to these target elements in the presence of E2 as previously reported [25, 26], and the timing of the initial peak of ERα association was different from the ones in these studies, but this may be owing to the different experimental conditions (e.g., lack of α-amanitin synchronization in our assays).

**Fig. 3:**
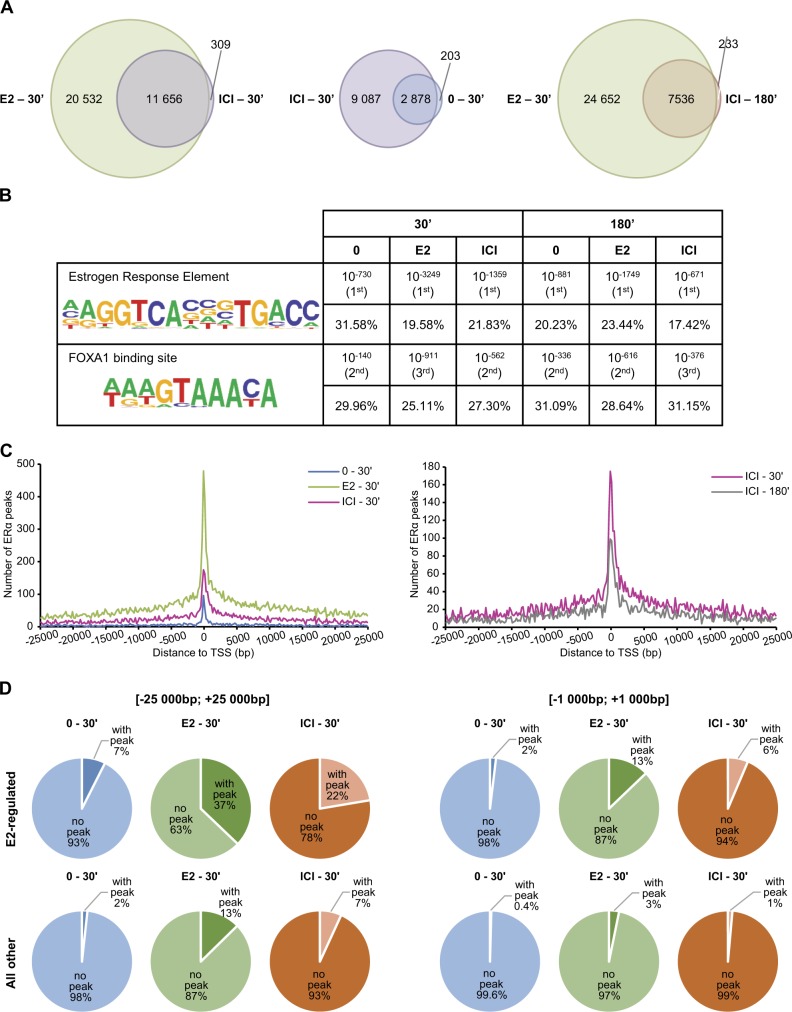
Genome-wide analysis supports transient DNA binding of ICI182,780-liganded ERα to a subset of E2-induced sites. ChIP-Seq was performed with an antibody against ERα on MCF-7 cells treated with estradiol (E2, 5 nM) or ICI182,780 (ICI, 100 nM) for the indicated time points (minutes) (*N* = 3). **a** The overlap between the ERα ChIP-Seq peaks for the indicated treatment conditions is shown using Venn diagrams. **b** Transcription factor motif enrichment analyses were performed using HOMER. The two top-ranking enriched motifs in the 30 min ICI condition are shown along with their *P* value and rank, and with the percentage of target sequences containing these motifs, for each treatment condition. **c** The number of called peaks in a (− 25 kb; + 25 kb) window centered on gene TSS was plotted for the indicated treatment conditions. **d** Gene annotations from our RNA-Seq data set were divided into two categories: “E2-regulated” (significantly regulated (*q* value < 0.05) after 16 h of E2 treatment) and “All other” (all other gene annotations). The percentage of gene annotations with at least one ERα ChIP-Seq peak after 30 min of treatment (0, E2, ICI) in various windows centered on their TSS was determined for each category

We further performed ChIP-Seq analyses of ERα binding to DNA following 30 min or 3 h treatment with E2 or ICI182,780. In the unsupervised PCA analyses of peaks called by model-based analysis of ChIP-Seq (MACS) using log2-normalized counts (Suppl. Figure 3A, B), samples treated for 30 min by E2 or ICI182,780 clustered separately from non-treated samples, whereas the samples treated for 180 min were less distinguishable. Differential analysis of peak numbers indicated at the genome-wide scale a significant recruitment of ERα to target chromatin regions after 30 min of treatment with E2 (32,188 peaks) or ICI182,780 (11,965 peaks) compared with the absence of treatment (3081 peaks, Fig. 3a and Suppl. Table 2). In total, 97% of ICI182,780 peaks overlapped with E2 peaks at this time point (Fig. 3a). After 3 h of ICI182,780 treatment, however, fewer ERα-binding events were detected (7769 peaks, Fig. 3a and Suppl. Table 2), still essentially corresponding to peaks observed with E2 at 30 min (97% overlap). These results indicate association of ERα with DNA in the presence of ICI182,780 at a subset of the sites bound in the presence of E2 at 30 min, with decreased binding at 3 h compared with 30 min (Fig. 3a).

Enrichment analysis for known transcription factor motifs returned EREs and FOXA1-binding sites as the top hits in the presence of E2, consistent with results from previous ERα ChIP-Seq studies [31], as well as in the presence of ICI182,780 (Fig. 3b). This suggests that ERα recruitment at FOXA1-binding sites is independent of agonist-specific protein–protein interactions engaged by the receptor. ERα peak location analyses also revealed very similar profiles regardless of treatment conditions (Fig. 3c and Suppl. Figure 3C). Heatmaps of ChIP-Seq binding for ERα in the presence of E2 and ICI182,780 at 30 min at regions containing EREs (3260 peaks) are consistent with intermediate levels of binding for ICI182,780-treated vs. 0- and E2-treated samples at 30 min, and decreased binding intensity for ICI182,780-treated samples at 3 h vs. 30 min (Suppl. Figure 3D). Notably, ERα-binding events were enriched in the flanking regions (− 25 to + 25 kb from TSS) of E2-regulated genes compared with all other genes in the presence of ICI182,780 (22% vs. 7%), as well as E2 (37% vs. 13%), and vehicle (7% vs. 2%) (Fig. 3d). When considering a narrower window around the TSS (− 1 to + 1 kb), this enrichment was even more marked (four- to sixfold, Fig. 3d), compatible with a role for ERα in the transcriptional regulation of neighboring genes. Together, these results confirm that the near absence of partial agonist activity observed with ICI182,780 compared with E2 (Fig. 1b) does not result from a lack of initial recruitment of ERα to DNA.

### Rapid loss of binding to DNA precedes loss of ERα protein and is not affected by proteasome inhibition

To test whether the loss of DNA binding in the presence of ICI182,780 was due to delocalization of endogenous ERα in MCF-7 cells, as previously reported for mouse ERα in transfected COS-1 cells [32], we performed immunofluorescence after 30 min, 1 h, and 3 h of ICI182,780 treatment, corresponding to maximal, weak, and no binding of ERα to DNA, respectively. No significant difference in ERα nuclear localization was observed over this time course of treatment compared with no treatment (Suppl. Figure 4). Thus, loss of ERα binding to DNA in MCF-7 cells cannot be explained by an increased rate of export from the nucleus in the presence of ICI182,780.

**Fig. 4:**
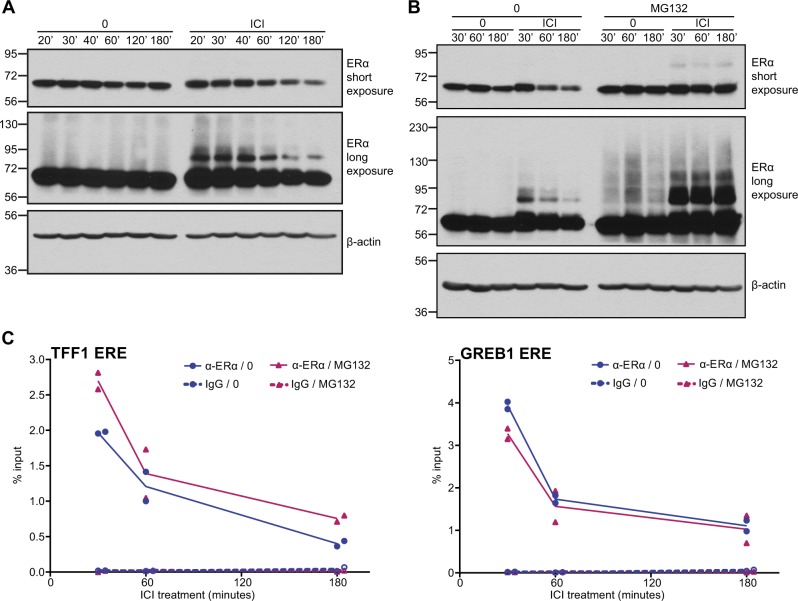
Loss of binding to DNA occurs irrespective of ERα degradation. **a** MCF-7 cells were treated with ICI182,780 (ICI, 100 nM) for the indicated time points (minutes). Whole cell extracts were resolved by SDS-PAGE, and ERα and β-actin levels were assessed by Western analysis. Two different film exposures are shown to reveal ERα degradation (short exposure) and receptor modification (long exposure). A representative experiment is shown (*N* = 3). **b** MCF-7 cells were pre-treated with the proteasome inhibitor MG132 (10 μM) for 2 h then treated with ICI (100 nM) for the indicated time points (minutes). Whole cell extracts were resolved by SDS-PAGE, and ERα and β-actin levels were assessed by western analysis. Two different film exposures are shown to reveal ERα degradation (short exposure) and receptor modification (long exposure). A representative experiment is shown (*N* = 3). **c** MCF-7 cells were treated as in **b**. ERα binding to target gene regulatory regions was determined by ChIP-qPCR. Graphs compare ERα binding in the presence of ICI with or without MG132 pre-treatment. Data points from two independent experiments are represented. *P* values in a two-tailed *t* test do not support significantly different results

Past studies have shown that SERD activity, associated with pure AEs, leads to increased degradation of ERα via the ubiquitin—proteasome pathway [21, 22, 24, 33]. To compare the kinetics of ERα binding to DNA to those of receptor degradation, we monitored the steady state levels of ERα in MCF-7 cells at different times after ICI182,780 addition. Concordant with previous results [24, 33], loss of receptor was not detectable until 60 min of ICI182,780 treatment (total extraction buffer; Fig. 4a, top panel) and increased progressively thereafter, whereas decreased binding to DNA was already observed at 40 min (Fig. 2), suggesting that loss of ERα binding to DNA precedes receptor degradation.

To more directly evaluate the impact of ERα degradation on its ability to bind DNA, we pre-treated MCF-7 cells with the proteasome inhibitor MG132 for 2 h, and performed ChIP assays following ICI182,780 treatment for 30 min, 1 h, or 3 h. ERα protein levels were assessed at the same time points by western analysis of whole cell extracts. MG132 pre-treatment stabilized ERα levels (Fig. 4b, top panel) without affecting kinetics of release of receptor from the *TFF1* and *GREB1* EREs (Fig. 4c), suggesting that the loss of binding to DNA is independent of ERα degradation.

### SUMO2/3 marks coincide with ERα binding to DNA in the presence of pure AEs

ERα modification by ubiquitination and SUMOylation is induced by pure AEs/SERDs prior to receptor degradation [22, 24]. Modified forms of ERα were detected at 20 min after ICI182,780 addition, indicating that these modifications precede loss of receptor binding to DNA. The amount of modified receptor appeared stable at 40 min, then decreased from 60 to 180 min parallel to the loss of unmodified receptor (Fig. 4a, middle panel). The discrete modified bands observed in the presence of ICI182,780 and the absence of MG132 in MCF-7 cells were previously shown by immunoprecipitation (IP) with an ERα antibody and blotting with SUMO antibodies to correspond to SUMOylated forms of ERα [24], which, as reported for other proteins [34], represent a small fraction of total ERα at any given time. Conversely, blotting with an ERα antibody after an IP with a SUMO2/3 antibody led to the detection of both unmodified and modified forms of endogenous ERα in the ICI182,780-treated, but not the untreated samples (Suppl. Figure 5A). Detection of unmodified ERα may be due to deSUMOylation during the IP procedure, or possibly also to non-covalent interactions between ERα and SUMO2/3 moieties taking place through putative SUMO-interacting motifs [34]. A more complex ladder of modified ERα forms can be observed both in the absence and presence of ICI182,780 in MG132-treated MCF-7 cells (Fig. 4b, middle panel), consistent with poly-ubiquitinated forms being present under basal conditions and induced by pure AEs/SERDs [22, 26].

**Fig. 5:**
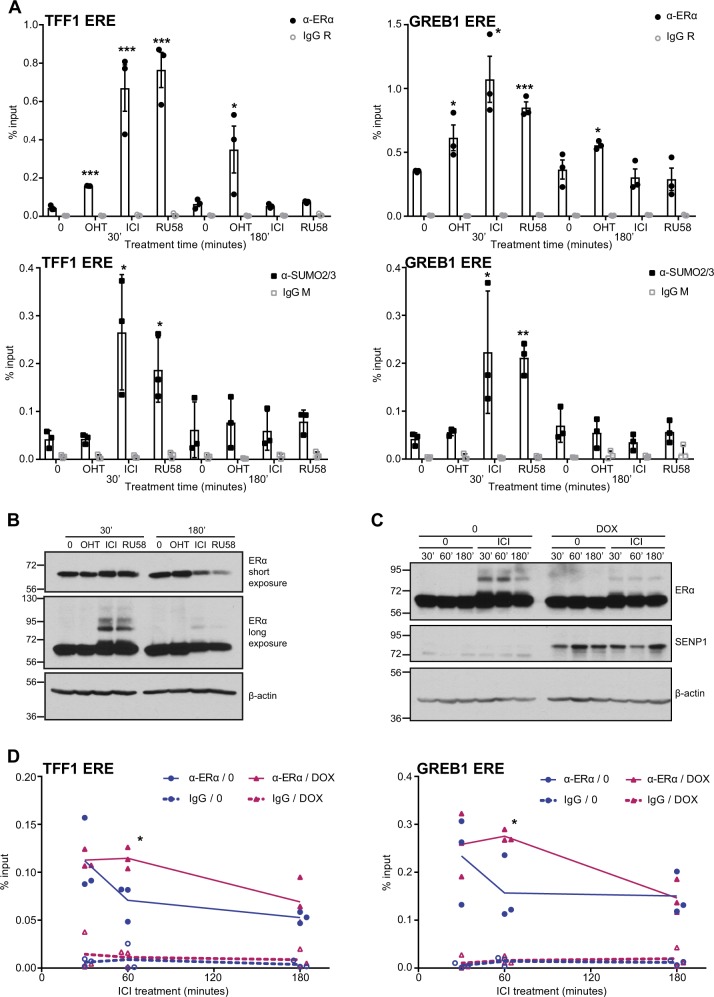
SUMOylation contributes to the rapid loss of ERα from DNA. **a** Binding of ERα or SUMO2/3 to E2 target gene regulatory regions was determined by ChIP-qPCR in MCF-7 cells treated with 100 nM of 4-hydroxytamoxifen (OHT), ICI182,780 (ICI), or RU58668 (RU58) for the indicated time points. Data points from three independent experiments, as well as means ± SEM, are represented. Asterisks denote significance (one-tailed *t* test, AE vs. 0): * *P* value < 0.05; ** *P* value < 0.005; *** *P* value < 0.0005. **b** ERα and β-actin levels were assessed by western analysis in whole cell extracts from MCF-7 cells treated as in **a**. Two different film exposures are shown to reveal ERα degradation (short exposure) and receptor modification (long exposure). A representative experiment is shown (*N* = 3). **c** ERα, SENP1, and β-actin levels were assessed by western analysis in whole cell extracts from MCF-7 Tet-ON SENP1-FLAG cells induced or not with doxycycline (DOX, 3 μg/mL) for 24 h, and subsequently treated with ICI (100 nM) or vehicle only (0) for the indicated time points (minutes). A representative experiment is shown (*N* = 3). **d** ERα binding to target gene regulatory regions was determined by ChIP-qPCR in MCF-7 Tet-ON SENP1-FLAG cells treated as in **c**. Graphs compare ERα binding in the presence of ICI with or without DOX induction. Data points from three independent experiments are represented. Asterisks denote significance (one-tailed *t* test, 0 vs. DOX): * *P* value < 0.05

To investigate the role of SUMOylation in the loss of binding of ERα to DNA, we compared association to DNA of ERα and SUMO2/3 (SUMO2 being the most expressed paralog at the RNA level in MCF-7 cells, Suppl. Figure 5B), following treatment with the SERM OHT or the pure AEs ICI182,780 or RU58668. ChIP after 30 min of treatment revealed significant binding of ERα to EREs upstream of *TFF1*, *GREB1*, and *CTSD* with OHT, ICI182,780, and RU58668 compared with vehicle (Fig. 5a and Suppl. Figure 5C). At the same time, a significant increase in the SUMO2/3 ChIP signal was only detected for the ICI182,780 and RU58668 conditions at the studied EREs (Fig. 5a and Suppl. Figure 5C), consistent with the detection of modified forms of ERα for these pure AEs, but not for OHT, by immunoblotting (Fig. 5b). At 3 h of treatment, ERα was bound to DNA in the presence of OHT, but not of the pure AEs (Fig. 5a and Suppl. Figure 5C), despite reduced but still detectable overall levels of ERα (Fig. 5b). No significant increase in recruitment of SUMO2/3 to DNA was detected either in the presence of OHT or of pure AEs at this time (Fig. 5a and Suppl. Figure 5C). In contrast with observations at 30 min of ICI182,780 treatment on EREs upstream of *GREB1* and *CTSD*, association of SUMO2/3 with control regions in the *GREB1* and *CTSD* gene bodies, which do not overlap with EREs, was not increased at the same time point (Suppl. Figure 5D). However, ICI182,780 induced recruitment of SUMO2/3 moieties to several other EREs found within ERα ChIP-Seq peaks in the vicinity of genes regulated by E2 (Suppl. Figure 5E). Thus, SUMO2/3 recruitment as assessed by ChIP at ERα target regions correlates with the SUMOylation pattern and the ERα DNA-binding profile induced by pure AEs, suggesting that SUMOylated forms of ERα are associated with DNA at early time points.

**Fig. 6:**
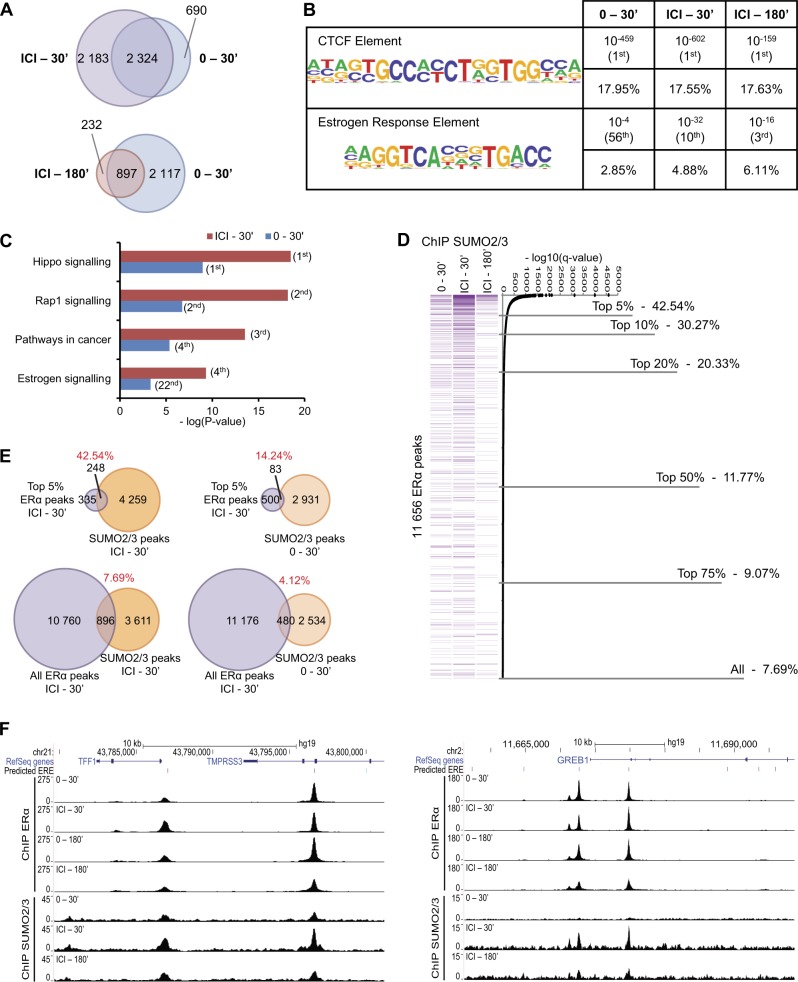
Enrichment of SUMO2/3 moieties at regions strongly bound by ERα in the presence of ICI182,780. ChIP-Seq was performed with an antibody against SUMO2/3 on MCF-7 cells treated with ICI182,780 (ICI, 100 nM) for the indicated time points (minutes) (*N* = 2). **a** The overlap between the SUMO2/3 ChIP-Seq peaks for the indicated treatment conditions is shown using Venn diagrams. **b** Motif enrichment analyses were performed using HOMER. Enrichment in CTCF and ERE motifs is shown along with the *P* value, rank, and percentage of target sequences containing these motifs for each treatment condition. **c** KEGG pathway enrichment analyses were performed using HOMER. The Top four ranking pathways (ICI, 30′) are indicated along with their significance and rank for the different treatment conditions. **d** Overlap between ERα and SUMO2/3 peaks. ERα ChIP-Seq peaks (common between E2, 30′ and ICI, 30′) were ordered according to their -log10(*q* value) provided by MACS. The proportion of ERα peaks overlapping with SUMO2/3 ChIP-Seq peaks following treatment with ICI for 30 min is indicated. **e** The overlaps between the Top 5% ERα peaks (ICI, 30′) and SUMO2/3 peaks (0 or ICI, 30′) (top) or between all ERα peaks (ICI, 30′) and SUMO2/3 peaks (0 or ICI, 30′) (bottom) are indicated using Venn diagrams. **f** UCSC browser snapshots of ERα and SUMO2/3 ChIP-Seq peaks at EREs near E2 target genes *TFF1* and *GREB1* for the vehicle and ICI conditions

We next performed ChIP-Seq analyses to map the genome-wide distribution of SUMO2/3 marks following ICI182,780 treatment in MCF-7 cells. In a PCA analysis of peaks called by MACS based on log2-normalized counts (Suppl. Figure 6A, Supplementary Material and Methods), samples grouped by treatment condition. ICI182,780 treatment for 30 min led to an increase in SUMO2/3 peak numbers (4507 vs. 3014), followed by a reduction at 3 h (1129 peaks, Fig. 6a and Suppl. Table 2).

SUMO2/3 peak location analyses revealed similar profiles in all conditions (Suppl. Figure 6B), with a higher proportion of proximal peaks and fewer gene-associated peaks compared with ERα-binding distributions (Suppl. Figure 3C). Interestingly, whereas CTCF elements remained the top enriched motif in SUMO2/3 peaks regardless of treatment conditions, EREs figured among the top 10 enriched motifs in peaks observed after ICI182,780 treatment, but not in the vehicle control (Fig. 6b). Furthermore, several motifs enriched in ERα peaks (e.g.: Jun, AP1, and FOXA2 elements) were also found to be enriched in SUMO2/3 peaks after 30 min of ICI182,780 treatment (Suppl. Table 3), suggesting an overlap of ERα and SUMO2/3 presence on different DNA motifs. Moreover, pathway enrichment analysis for SUMO2/3 peaks returned estrogen signaling at a much higher rank in the ICI182,780-treated sample (30 min) than for the control sample (Fig. 6c). Interestingly, the proportion of ERα peaks (detected under both E2 and ICI182,780 treatment for 30 min) overlapping with SUMO2/3 peaks after 30 min of ICI182,780 treatment was highest for the ERα peaks with a strong confidence level (MACS -log10(*q* value)) (Fig. 6d, e). Indeed, 42.5% of the Top 5% ERα peaks overlapped with a SUMO2/3 peak in the ICI182,780-treated cells, compared with only 7.69% overlap for all ERα peaks (Fig. 6d, e). This represented a threefold enrichment compared with overlap with SUMO2/3 peaks in the absence of treatment for the Top 5% ERα peaks, vs. twofold for all ERα peaks (Fig. 6e). A heat map representation of SUMO2/3 binding for the Top 5% ERα peaks indicated an overall increased SUMO2/3 binding in the ICI182,780-treated samples at 30 min compared with control samples and ICI182,780-treated samples at 3 h (Suppl. Figure 6C; note that although regions were selected based on strong ERα binding, correlation between biological replicates for association with SUMO at these peaks was ≥ 0.65 (Spearman) or 0.99 (Pearson) for each treatment, not shown). The regions with highest overall SUMO counts at the top of the graph correspond to regions of higher background also in the input sequences, and likely reflect regions of focal amplification; these regions were not removed as they also contain detectable ERα and SUMO2/3 peaks. Finally, UCSC browser examination of several E2 target genes (*TFF1*, *GREB1*, *CTSD*, *CDH26*) confirmed an increased SUMO2/3 ChIP-Seq signal at DNA regions also bound by ERα following 30 min of ICI182,780 treatment (Fig. 6f and Suppl. Figure 6D), in keeping with observations by ChIP-qPCR (Fig. 5a and Suppl. Figure 5C-E).

### SUMOylation of ERα contributes to the rapid loss of binding to DNA

To further investigate the impact of SUMOylation of ERα on its association with DNA, we generated an MCF-7 cell line stably expressing the deSUMOylase SENP1 from an inducible Tet-ON system. Western analysis of these cells showed that SENP1 overexpression following a 24 h induction with doxycycline (DOX) resulted in decreased ERα modification levels in cells treated with ICI182,780 for 30 min and 1 h compared with control samples (Fig. 5c). ChIP experiments on EREs upstream of *TFF1* and *GREB1* under these conditions revealed that ERα binding to DNA was significantly increased in the presence of ICI182,780 at 1 h in DOX-treated cells compared with non-induced cells (Fig. 5d). At 3 h, binding levels were similar in both DOX-induced and non-induced samples. Note that ERα modification levels were similar in the ± DOX conditions at this time by western analysis (Fig. 5c). SUMOylation thus appears to contribute to the rapid loss of ERα binding to DNA.

### ICI182,780 induces rapid chromatin closure at estrogen target genes

**Fig. 7:**
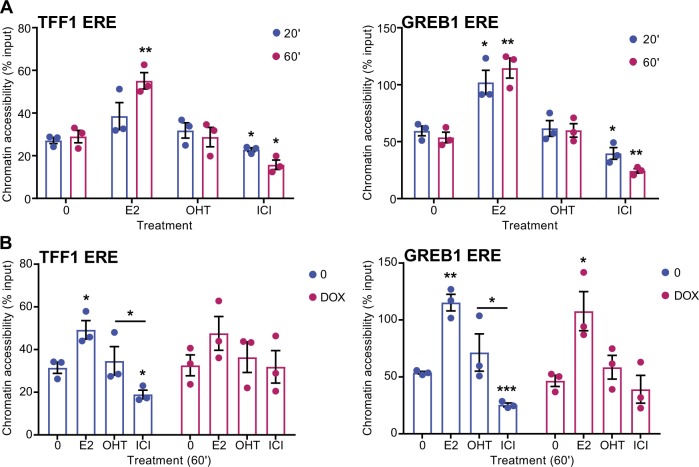
ICI182,780 induces rapid chromatin closure at estrogen target genes. **a** Levels of accessible chromatin at ER target regions were assessed by formaldehyde-assisted isolation of regulatory elements (FAIRE) in MCF-7 cells treated with estradiol (E2, 5 nM), 4-hydroxytamoxifen (OHT, 100 nM), ICI182,780 (ICI, 100 nM), or vehicle only (0) for the indicated time points (minutes). Data points from three independent experiments, as well as means ± SEM, are represented. **b** Levels of accessible chromatin at ER target regions were assessed by FAIRE in MCF-7 Tet-ON SENP1-FLAG cells induced with doxycycline (DOX, 3 μg/mL) for 24 h, and subsequently treated with E2 (5 nM), OHT (100 nM), ICI (100 nM), or vehicle only (0) for 1 h. Data points from three independent experiments, as well as means ± SEM are represented. Asterisks denote significance (one-tailed *t* test, vs. 0): * *P* value < 0.05; ** *P* value < 0.005; *** *P* value < 0.0005

To investigate the impact of pure AE treatment on chromatin state at ER target regions, we performed formaldehyde-assisted isolation of regulatory elements (FAIRE), which enables isolation of nucleosome-depleted regions permissive to transcription factor and cofactor binding [35]. Following treatment with the agonist E2, chromatin at EREs located upstream of target genes *TFF1*, *GREB1*, and *CTSD* was significantly more accessible than in the absence of ligand at 1 h, consistent with recruitment of ERα and co activators at these sites. OHT did not alter the chromatin state at these EREs compared with the vehicle condition, whereas ICI182,780 treatment markedly decreased accessibility of these regions at 1 h (Fig. 7a and Suppl. Figures 7A) or 3 h (data not shown) after ligand addition. Strikingly, decreased accessibility was already observed, albeit at a lower level, 20 min after ICI182,780 addition, before the peak of ERα binding and its subsequent release from DNA. Chromatin accessibility was also decreased at 20 min in control regions in the *GREB1* and *CTSD* gene bodies, although not in a statistically significant manner (Suppl. Figure 7C), possibly reflecting their decreased transcription in the presence of ICI182,780. Finally, FAIRE-qPCR on additional EREs validated the observed reduction of chromatin accessibility in the presence of ICI182,780 compared with the other treatments (Suppl. Figure 7D). These results suggest that ICI182,780 induces a progressive chromatin shut-off at promoters and enhancers of E2 target genes, leading to a more complete repression of ERα transcriptional activity compared with OHT.

To test whether SUMOylation plays a role in chromatin closure in the presence of ICI182,780, we performed FAIRE experiments in the Tet-ON SENP1 cell line following 1 h of treatment. These assays revealed that SENP1 overexpression attenuated chromatin closure in the presence of ICI182,780 at the *TFF1*, *GREB1*, and *CTSD* EREs compared with untreated controls or to OHT (Fig. 7b and Suppl. Figure 7B). Together, these results suggest that SUMOylation contributes to the chromatin closure induced by ICI182,780, although other factors, such as altered cofactor recruitment, may also play a role.

As all above-described experiments were performed in estrogen-depleted media, we sought to determine whether the main conclusions were reproducible in MCF-7 cells grown in complete media containing estrogenic factors, these latter conditions being closer to the tumor environment encountered in the clinic. ChIP-qPCR confirmed that ERα binding to DNA was significantly increased following 1 h of ICI182,780 treatment compared with vehicle, but not at 3 h (Suppl. Figure 8A). Moreover, chromatin closure at EREs was also observed after 3 h of ICI182,780 treatment in MCF-7 cells grown in complete media (Suppl. Figure 8B).

### Downregulation of SUMOylation alleviates repression of ERα transcriptional activity by ICI182,780

**Fig. 8:**
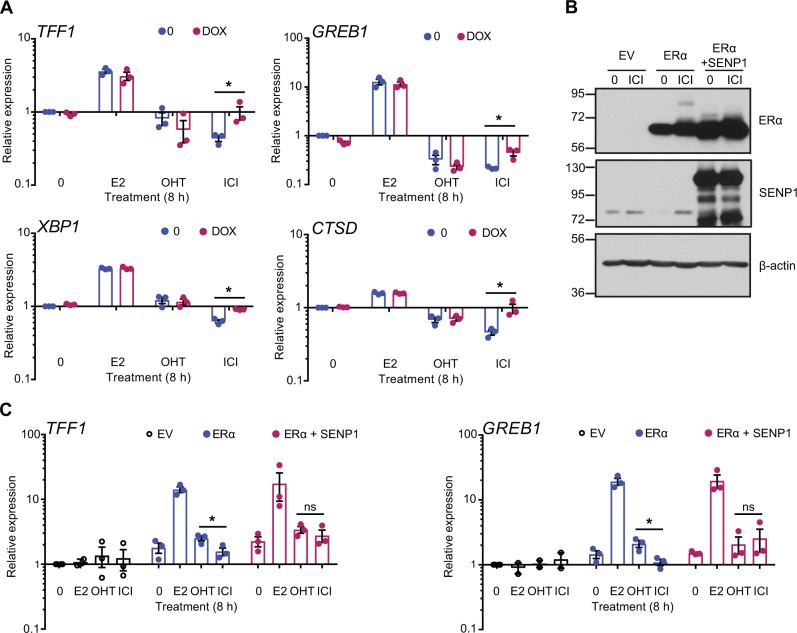
Inhibiting SUMOylation before ICI182,780 treatment partially de-represses ERα transcriptional activity. **a** mRNA levels of E2 target genes were determined by RT-qPCR in MCF-7 Tet-ON SENP1-FLAG cells induced or not with doxycycline (DOX, 3 μg/mL) for 24 h, and subsequently treated with estradiol (E2, 5 nM), 4-hydroxytamoxifen (OHT, 100 nM), ICI182,780 (ICI, 100 nM), or vehicle only (0) for 8 h. Data points from three independent experiments, as well as means ± SEM, are represented. Asterisks denote significance (one-tailed *t* test): * *P* value < 0.05. **b** ERα, SENP1, and β-actin levels were assessed in whole cell extracts from SK-BR-3 cells transiently transfected with plasmids coding for ERα and for GFP-tagged SENP1 and treated with ICI (1 µM) or vehicle only for 30 min. A representative experiment is shown (*N* = 3). EV: empty vector. **c** mRNA levels of E2 target genes were determined by RT-qPCR in SK-BR-3 cells similarly transfected and treated with E2 (5 nM), OHT (1 µM), or ICI (1 µM) for 8 h. Data points from three independent experiments, as well as means ± SEM, are represented. Asterisks denote significance (one-tailed *t* test): * *P* value < 0.05

To test whether downregulation of SUMOylation in the presence of ICI182,780 leads to an altered regulation of estrogen target gene expression, we treated the MCF-7 Tet-ON SENP1 cells with E2, OHT or ICI182,780 for 8 h and quantified the expression of direct E2 target genes *TFF1*, *GREB1*, *XBP1*, and *CTSD*. Although gene expression levels did not change in cells overexpressing SENP1 (DOX, magenta) vs. non-induced cells (0, blue) treated with vehicle, E2 or OHT, repression of transcription by ICI182,780 was significantly attenuated for all tested genes (Fig. 8a). For *TFF1*, *XBP1*, and *CTSD*, mRNA levels in the DOX + ICI182,780-treated cells were similar to those in the vehicle condition, whereas repression of transcription was only partially relieved in the case of *GREB1* (Fig. 8a). These results indicate that decreased SUMOylation of ERα reduces the antagonistic potential of ICI182,780.

To confirm these results in another BC cell model, we transiently co-transfected wild-type ERα with GFP-tagged SENP1 or its parental empty vector in the ER-negative SK-BR-3 cells (Fig. 8b). Treating ERα-transfected cells with E2 led to induction of target genes *TFF1* and *GREB1* (Fig. 8c). On both genes, ICI182,780 led to significantly stronger inhibition of gene expression than OHT (Fig. 8c). However, upon inhibition of ERα SUMOylation by SENP1 overexpression (Fig. 8b), ERα activity in the presence of ICI182,780 was de-repressed and became indistinguishable from the activity in the presence of OHT (Fig. 8c). Thus, downregulating SUMOylation diminished the antiestrogenic character of ICI182,780 in this cell model as well as in MCF-7 cells.

**Fig. 9:**
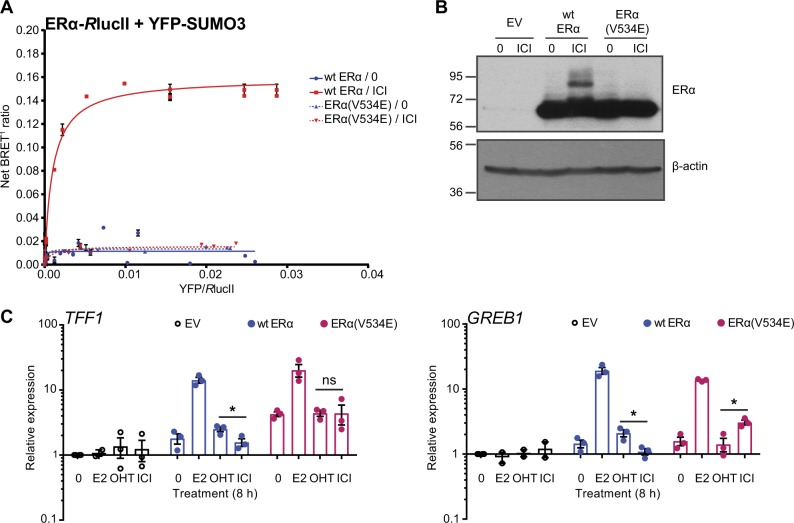
The ERα V534E mutation prevents SUMOylation and abrogates differential gene repression by ICI182,780 and OHT. **a** Interaction between ERα and SUMO3 was determined by BRET^1^ assays (*N* = 2) in HEK-293 cells transfected with a constant amount of wt or mutant ERα-*R*lucII expression vector and increasing amounts of YFP-SUMO3 plasmid, and treated with ICI182,780 (ICI, 1 µM) or vehicle only (0) for 1 h. **b** ERα and β-actin levels were assessed in whole cell extracts from SK-BR-3 cells transiently transfected with wt ERα, mutant receptor ERα(V534E), or empty vector (EV) and treated with ICI (100 nM) or vehicle only (0) for 30 min. A representative experiment is shown (*N* = 3). **c** mRNA levels of E2 target genes *TFF1* and *GREB1* were determined by RT-qPCR in SK-BR-3 cells similarly transfected and treated with E2 (5 nM), OHT (1 µM), ICI (1 µM), or vehicle only (0) for 8 h. Data points from three independent experiments, as well as means ± SEM, are represented. Asterisks denote significance (one-tailed *t* test): * *P* value < 0.05

Because overexpression of SENP1 leads only to partial suppression of SUMOylation (Fig. 5c) and may impact other chromatin-associated proteins than ERα, we sought to use ERα mutants affected in their capacity to be SUMOylated in the presence of pure AEs. Mutation of several characterized SUMOylation sites in ERα did not abrogate modification in the presence of ICI182,780 [24]. However, in a screen of ERα mutations identified in BC relapses after hormonal therapy, we found that mutation V534E prevented SUMOylation in the presence of pure AEs (El Ezzy et al., in preparation). Indeed, BRET assays between ERα coupled to *R*lucII and SUMO3 fused to yellow fluorescent protein (YFP) in transfected HEK-293 cells revealed a specific interaction between ERα and SUMO3 in the presence of ICI182,780 only for the wt receptor (Fig. 9a), whereas no SUMOylation of the V534E mutant could be detected in a time course of up to 4 h of treatment (Suppl. Figure 9). In addition, western analysis did not detect modified forms of the V534E mutant in transfected HEK-293 (not shown) or SK-BR-3 cells (Fig. 9b).

Transient transfection of wt or V534E mutant forms of the receptor in SK-BR-3 cells indicated that, contrary to other mutations associated with resistance to hormonal therapies, e.g., at positions Leu536, Tyr537, and Asp538 [36–39], V534E does not lead to constitutive activity of the receptor (Fig. 9c). However, transcriptional activity of this mutant in SK-BR-3 cells was significantly increased compared with wt ERα in the presence of ICI182,780 for *TFF1* and *GREB1* (Fig. 9c). Furthermore, contrary to what was observed with wt ERα, ICI182,780 did not repress transcription of these genes more efficiently than OHT with the V534E mutant (Fig. 9c).

Together, our results indicate that SUMOylation of ERα contributes to its transcriptional repression by pure AEs on endogenous target genes by inducing rapid loss of receptor binding to response elements at estrogen target genes.

## Discussion

Consistent with prior observations obtained with different culture conditions, time of exposure to AEs and gene expression profiling platforms [14, 15], our RNA-Seq profiling of ER-positive MCF-7 cells revealed varying degrees of partial agonist activity of OHT on almost half of E2 targets, whereas ICI182,780 was devoid of activity or opposed E2 effects on nearly all E2-regulated genes. We then explored reported mechanisms of action specific to pure AEs for their relevance to the increased transcriptional suppression of estrogen target genes in MCF-7 cells.

We did not detect differences in nuclear localization of the endogenous ERα during our experimental time frame in MCF-7 cells. This result contrasts with the observed alteration in nucleo-cytoplasmic shuttling of mouse ERα in transfected COS-1 cells [32], but is in agreement with differential extraction experiments in MCF-7 cells [24, 40], and with localization of transfected GFP-tagged ERα in various BC cell lines treated with ICI182,780 [41, 42].

Our observation of a transient phase of increased association of ERα to EREs between 20 and 40 min of treatment with pure AEs ICI182,780 and RU58668, followed by a marked loss of association with DNA at longer time points, reconciles previous apparently divergent reports [26, 27]. These results are also compatible with ERα binding to DNA in the presence of pure AEs in gel shift experiments [43], but suggest progressive exclusion of receptor from chromatin in pure AEs-treated MCF-7 cells. ERα degradation through the ubiquitin—proteasome pathway, which is induced by AEs with SERD activity, was not responsible for the observed loss of binding to DNA in the presence of ICI182,780, which precedes the decrease in overall ERα protein levels and happens irrespective of proteasome inhibition. Of note, saturation of the ERα degradation process was also found not to prevent transcriptional suppression of target gene *TFF1* by ICI182,780 in MCF-7 cells [44].

Building on our previous report that ERα SUMOylation is induced by pure AEs, but not by the SERM OHT [24], we investigated the impact of this post-translational modification on the ability of endogenous ERα to bind its target genomic regions and activate gene transcription in the MCF-7 BC cell line. We show that SUMO2/3 moieties were detected at EREs in the presence of pure AEs, but not OHT. In the absence of a specific antibody recognizing SUMOylated ERα, it is not possible to conclude that SUMO-modified proteins detected at EREs correspond specifically to modified forms of ERα. Receptor-associated cofactors and histones may be additional modification targets at ERα-bound DNA sites [45–48]. However, the SUMO marks detected at EREs disappeared with release of ERα from DNA, even though chromatin remains more closed in the presence of ICI182,780 than in basal conditions, suggesting that these marks are associated with DNA-bound ERα complexes rather than with histones assembled on EREs. The observations that modified ERα was detected as early as 20 min, i.e., before the peak of binding to DNA, and was found in both the chromatin-bound and the nuclear matrix fractions in MCF-7 cells at 1 h [24], are compatible with SUMOylated ERα being associated to DNA. SUMOylation of several general TFs or enhancer factors, including nuclear receptors, was previously reported to take place on DNA [49, 50] and to be associated with transcriptional repression [45, 46, 48, 51–55]. Accordingly, SUMO2/3 ChIP-Seq peaks were enriched in different transcription factor-binding sites, especially CTCF motifs. However, the enrichment in EREs was stronger after treatment with ICI182,780, consistent with increased SUMOylation of ERα on DNA. Possible reasons for the partial overlap between ERα peaks and SUMO2/3 peaks could include differences in SUMO2/3 association strength/kinetics at different ERα-bound regions, but also likely the lower number of SUMO2/3 peaks detected (~ 4500 SUMO2/3 peaks compared with ~ 12,000 ERα peaks in the presence of ICI182,780 at 30 min). In addition, the median peak height and highest peak for the SUMO2/3 data set (7.4 and 550 reads, respectively) were much lower than those observed for the ERα data set (11.4 and 4400 reads, respectively) in the presence of ICI182,780 (30 min). Finally, the stronger overlap with SUMO2/3 peaks observed with the top ERα peaks is consistent with the presence of SUMO2/3 marks on only a fraction of ERα bound at a specific site, resulting in better detection at DNA sites with a more stable association with ERα.

**Fig. 10:**
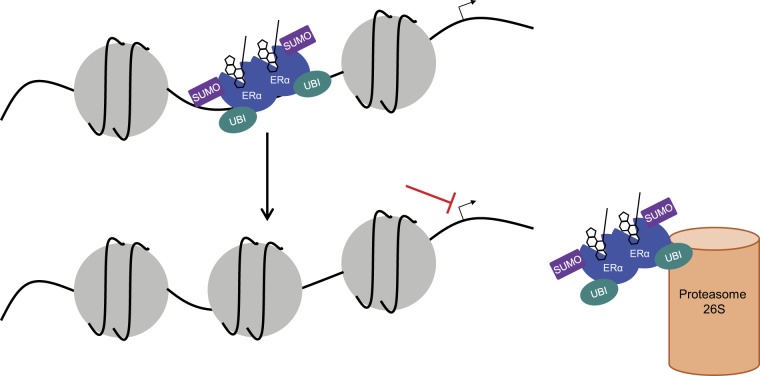
Proposed mechanism of action of pure AEs in ER-positive breast cancer cells. Pure AE treatment leads to transient binding of ERα to DNA and induces post-translational modification of the receptor by SUMO and ubiquitin. Chromatin at ER target regions is rapidly compacted, preventing re-association of ERα and efficiently repressing transcription. ERα is also progressively degraded by the proteasome, ensuring longer-term blockage of ER signaling by pure AEs

SUMO-dependent repression of androgen receptor and glucocorticoid receptor [56, 57] was shown to be mediated by the corepressor DAXX, which recognizes the modified receptors and, together with HDAC1, HDAC2, DNMT1, and ATRX, induces chromatin closure [58–60]. Similarly, we observed that ICI182,780, but not OHT, rapidly decreased chromatin accessibility at the regulatory regions of E2 target genes, correlating with induction of ERα SUMOylation. Specific corepressors recruited by SUMOylated ERα remain to be identified, but increased recruitment of the corepressor NCoR by ICI182,780-liganded ERα has been reported [61]. Of interest, SUMOylation of PPARγ and GR was shown to increase recruitment of NCoR/SMRT [62, 63], and SUMOylation of NCoR itself enhances its activity as a transcriptional repressor [47]. Receptor modification, in addition to altered conformation [64], may also contribute to the loss of coactivator recruitment [40]. We propose that ERα SUMOylation leads to release of ERα from DNA, either directly, or via the resulting rapid compaction of chromatin at the regulatory regions of E2 target genes. Parallel increased ubiquitination of ERα (facilitated or not by SUMOylation [65]) would lead to progressive degradation of the receptor in the nucleus [26, 33] (Fig. 10). Chromatin remodeling at ER target regions coupled with receptor degradation should increase both the efficacy and the duration of the antiestrogenic response, albeit in a manner that is likely reversible upon cessation of antiestrogenic treatment.

In agreement with this model, reducing ERα SUMOylation levels delayed the kinetics of loss of binding, reduced chromatin closing, and led to a partial de-repression of ERα transcriptional activity on its target genes in the presence of ICI182,780, resulting in similar degrees of antiestrogenicity for OHT and ICI182,780 in both ER-positive MCF-7 and transfected ER-negative SK-BR-3 cells. In addition, we found that the V534E mutation, characterized in metastatic tumors progressing after multiple lines of endocrine therapy including SERDs [36], prevents ERα SUMOylation and results in increased transcription of estrogen target genes in the presence of ICI182,780, without increasing basal activity. It will be interesting in the future to determine whether a specific pattern of mutations is observed after progression on treatment with pure AEs compared with other forms of hormonal therapies, reflecting the specific mechanisms of action of this class of molecules.

Together, our results demonstrate the relevance of SUMOylation to the suppression of ERα transcriptional activation properties by pure AEs in ER-positive BC cells, and provide a mechanistic framework for these effects via chromatin closure and suppression of subsequent ERα binding to its target response elements. Future studies will address the identity of cofactors recruited by pure AE-liganded ERα and the connection between SUMOylation and ubiquitination/degradation of ERα.

## Materials and methods

### Reagents and plasmids

MG132 (Millipore #474790 (Etobicoke, ON, Canada)), DOX (Sigma D-9891 (Oakville, ON, Canada)), 17β-estradiol (Sigma E2758), (Z)-4-hydroxytamoxifen (ThermoFisher #341210 (Pittsburgh, PA, USA)), ICI182,780 (Abcam ab120131 (Toronto, ON, Canada)) and RU58668 (ThermoFisher #3224) were used to treat cells. GFP-SENP1 was a gift from Dr. M.J. Matunis (John Hopkins University) and the pSG5-ERα plasmid for wt ERα was from Dr. P. Chambon (Université de Strasbourg). The V534E mutant was generated by site-directed mutagenesis. YFP-SUMO3 was a gift from Dr. M. Dasso (National Institutes of Health, Bethesda, MD). ERα-*R*lucII was generated by PCR amplification of ERα complementary DNA (cDNA). The PCR product was cloned between the *NheI* and *BamHI* restriction sites in the pcDNA3-*R*lucII plasmid (gift from Dr. M. Bouvier, IRIC), in order to fuse *R*lucII to the C-terminus of ERα.

### Cell culture

Cells were purchased from ATCC (Manassas, VA, USA) and were regularly tested for mycoplasma contamination. MCF-7 cells were maintained at 37 °C, 5% CO_2_ in Alpha MEM (Wisent 310–011 (St-Bruno, QC, Canada)) supplemented with 10% fetal bovine serum (FBS) (Sigma F1051), 1% l-glutamine (Wisent 609–065), and 1% penicillin–streptomycin (Wisent 450–201). SK-BR-3 and HEK-293 cells were maintained at 37 °C, 5% CO_2_ in Dulbecco's modified eagle medium (DMEM) (Wisent 319–005) supplemented with 10% FBS and 1% penicillin–streptomycin. Three days before experiments, cells were switched to phenol red-free DMEM (Wisent 319–050) containing charcoal-stripped FBS, 2% l-glutamine and 1% penicillin–streptomycin.

### Cell transfection

SK-BR-3 cells were electroporated with 2 µg of ERα expression plasmid and 6 µg of SENP1 expression plasmid per 5 × 10^6^ cells at 240 V and 950 µF (Gene Pulser® II, Bio-Rad (Mississauga, ON, Canada)). Culture media was changed 24 h post electroporation and cells were treated and collected 48 h post transfection. For BRET experiments, HEK-293 cells were seeded in 24-well plates (1.5 × 10^5^ cells/well) and co-transfected the next day with a constant amount of plasmid expressing ERα-*R*lucII and increasing amounts of the YFP-SUMO3 expression vector (ratio 1:5 DNA:polyethylenimine). The following day, HEK-293 cells were treated and processed for BRET assays.

### RNA extraction and RNA-Seq

Cell pellets were lysed with QIAzol (QIAgen 79306 (Toronto, ON, Canada)) and RNA was extracted per manufacturer’s instructions. Libraries were prepared with the KAPA Stranded RNA-Seq Library Preparation Kit (Roche (Laval, QC, Canada)) and samples were sequenced with the Illumina HiSeq2000 platform (Victoria, BC, Canada). Gene expression was computed with Kallisto [28] with default parameters (100 bootstraps) on the reference genome GRCh38 with the annotation of Ensembl v85 (with cDNA and RNA). Differentially expressed gene analyses were performed with Sleuth, an R package that implements statistical algorithms (Wald test) for differential analyses that leverage the bootstrap estimates [29]. A Log2 fold-change was calculated from the mean TPM value of each group for each Kallisto bootstrap and the reported value is the median of all of them. Script is available at https://github.com/maderlab/Oncogene2018-scripts.

### Reverse transcription, qPCR

RNA was reverse transcribed with RevertAid H Minus Reverse Transcriptase (ThermoFisher EP0451). cDNA was quantified by qPCR (Light Cycler 480) with Universal Probe Library (UPL) assays (Suppl. Table 4). Results were analyzed by the ΔΔC_t_ method using two endogenous control genes (*RPLP0* and *TBP*). For the MCF-7 assays (Fig. 1c), samples were normalized to the “Vehicle” condition. For the MCF-7 Tet-ON SENP1 assays (Fig. 8a), samples were normalized to the “No DOX, Vehicle” condition. For the SK-BR-3 assays (Figs. 8C and 9C), samples were normalized to the “Empty Vector, Vehicle” condition.

### ChIP

ChIP was performed as described [66] from MCF-7 cells treated with ERα ligands for various times with the following antibodies: ERα HC-20 (Santa Cruz Biotechnology sc-543 (Dallas, TX, USA)), rabbit IgG (Cedarlane 011-000-003 (Burlington, ON, Canada)), SUMO2/3 (Cedarlane M114-3), or mouse IgG (Cedarlane 015-000-003). The abundance of immunoprecipitated DNA fragments was quantified by qPCR (Light Cycler 480, Roche) with UPL assays (Roche) (Suppl. Table 5). Results were analyzed by the Percent Input Method.

### ChIP-Seq

Libraries were prepared with the KAPA DNA HyperPrep Library Kit (Roche), and samples were sequenced with the Illumina NextSeq500 platform (Flowcell High Output (400 M fragments)—150 cycles paired-end read). Analyses were performed with a pipeline developed at the McGill University and Génome Québec Innovation Centre (MUGQIC), as part of the GenAP project available at https://bitbucket.org/mugqic/mugqic_pipelines.

### Western analysis

Whole cell extracts were prepared as described [24] using a lysis buffer containing 50 mM Tris-HCl pH 7.5, 150 mM NaCl, 5 mM ethylenediaminetetraacetic acid, 2% sodium dodecyl sulphate, 0.5% Triton, 1% NP40. A total of 30 μg of samples were resolved by sodium dodecyl sulphate-polyacrylamide gel electrophoresis (8% acrylamide). Antibodies ERα 60 C (Millipore 04-820), β-actin (Sigma A5441), SENP1 C-12 (Santa Cruz Biotechnology sc-271360), horseradish peroxidase (HRP)-conjugated anti-rabbit (Cedarlane 111-035-003) and HRP-conjugated anti-mouse (Cedarlane 115-035-003) were used.

### FAIRE

FAIRE was performed as described [67] with slight modifications. After extraction, DNA was precipitated with two volumes of 95% ethanol, 0.3 M sodium acetate pH 5.2 and 20 μg/mL of glycogen (ThermoFisher #R0551) at − 80 °C. Samples were submitted to RNAse A (BioShop #RNA675.100 (Burlington, ON, Canada)) and proteinase K (ThermoFisher EO0491) digestions (30 min at 37 °C and 1 h at 55 °C, respectively) before reversing crosslink at 65 °C overnight. DNA was purified on EZ-10 columns (BioBasic (Markham, ON, Canada)). The abundance of soluble DNA fragments was quantified by qPCR (Light Cycler 480) with UPL assays (Suppl. Table 5). Results were analyzed by the Percent Input Method.

### BRET assays

Cells were detached using phosphate-buffered saline, re-plated in white 96-well plates (ThermoFisher 07-200-628) and supplemented with Coelenterazine H (10 µM, Nanolight Technology (Pinetop, AZ, USA)). Readings were collected using a multidetector plate reader (MITHRAS LB940, Berthold (Bad Wildbad, Germany)) with sequential integration of signals in the 480 nm and 530 nm windows, for luciferase and YFP light emissions, respectively. The BRET signal (530/480, BRET^1^) was determined by calculating the ratio of the light intensity emitted by the YFP fusion over that emitted by the *R*lucII fusion [24]. Values were corrected by subtracting the background BRET^1^ signal (*R*lucII fusion expressed alone). BRET^1^ ratios were expressed as a function of the [YFP]/[*R*lucII] expression ratio, estimated by measurement of total fluorescence and luminescence. Total fluorescence was determined with a microplate reader (FlexStation II, Molecular Devices (Sunnyvale, CA, USA)) using an excitation filter at 485 nm and an emission filter at 535 nm.

## Electronic supplementary material

Supplementary Materials and Methods, References and Figure Legends

Supplementary figures

Supplementary Table 1

Supplementary Tables 2–5
