# Supplementary material for: Role of SUMOylation in differential ERα transcriptional repression by tamoxifen and fulvestrant in breast cancer cells
Source: Oncogene. 2018 Sep 6;38(7):1019–37. doi: 10.1038/s41388-018-0468-9 (PMC6514857; doi:10.1038/s41388-018-0468-9)
Supplement: Supplementary file 1 — Supplementary Materials and Methods, References and Figure Legends [file 41388_2018_468_MOESM1_ESM.pdf]

SUPPLEMENTARY MATERIAL AND METHODS

Cell proliferation assays. MCF-7 cells were seeded in estrogen-depleted media in black 96-well plates with clear bottoms (ThermoFisher 07-200-588) at 2 000 cells/well. The next day (day 0), media was discarded, and cell numbers were assessed by an alamarBlue® (ThermoFisher DAL1100) cell viability assay (3 h incubation with reagent at 1:50 in fresh media), using a microplate reader (FlexStation II) with an excitation filter at 531 nm and an emission filter at 590 nm. Cells were then incubated with 5 nM E2, 100 nM OHT or 100 nM ICI182,780, and alamarBlue® assays were performed on days 2, 4, 6 and 8.

ChIP-Seq. Reads were trimmed from the 3' end to have a phred score of at least 30. Illumina sequencing adapters were removed from the reads, and all reads were required to have a length of at least 50 bp. Trimming and clipping were performed using Timmomatic *(1)*. The trimmed reads were mapped onto the hg19 reference genome using the aligner BWA *(2)*, which creates a Binary Alignment Map file (.bam). All readset BAM files from the same sample were merged into a single global BAM file using Picard (http://broadinstitute.github.io/picard/). BAM files were sorted and indexed using SAMtools (http://samtools.sourceforge.net/). Duplicate reads (marked using Picard) were removed from subsequent analyses. Reads from biological replicates were merged for use in peak calling and motif analyses. BigWig coverage tracks were generated from the aligned reads using deepTools2 *(3)*. The coverage was calculated as the number of reads per 50 bp bin and normalized by 1x sequencing depth (effective hg19 genome size = 2 451 960 000) with a maximum fragment length set at 600 bp. Peaks were called using MACS software

(https://github.com/taoliu/MACS) and annotated with HOMER (http://homer.ucsd.edu/homer/) using RefSeq annotations. Motif enrichment analyses were performed with HOMER. Peak overlap was determined using the Intersect tool from Galaxy (https://usegalaxy.org/). An overlap was considered when intervals intersected for at least 1 bp.

Downstream statistical analyses were performed using R and Bioconductor packages *(4)* and the deepTools2 suite *(3)* using a script available at https://github.com/maderlab/Oncogene2018-scripts. Specifically, we used DiffBind R package (version 2.4.8) *(5)* for exploratory analyses of the ChIP-seq data to conduct the principal component analysis of peaks called by MACS in each sample using the default settings. This analysis is based on TMM normalized (using edgeR *(6,7)*), using ChIP read counts minus control read counts and full library size. The final peak sets were obtained by pooling biological replicates in the MACS analyses.

Tag density heatmaps were plotted using computeMatrix and plotHeatmap functions from the deepTools2 suite as described in https://github.com/maderlab/Oncogene2018-scripts/blob/master/scripts/run-all-ChIPseq-analysis.Rmd

Immunofluorescence. MCF-7 cells were seeded in glass bottom microwell dishes (MatTek P35GCOL-0-14-C (Ashland, MA, USA)) at $2.75x10^5$ cells/well. The next day, cells were treated, then fixed with 3% paraformaldehyde for 30 min and permeabilized with a solution of 0.2% BSA, 0.3% Triton in 1X PBS for 30 min. Cells were incubated overnight at 4°C with the primary anti-ERα 60C antibody (Millipore 04-820; 1:1 000 in a solution of 1% BSA, 0.3% Triton in 1X PBS). The next day, cells were washed 3 times with a solution of 3% BSA, 0.05% Tween 20 in 1X PBS. An Alexa Fluor® 594 goat anti-rabbit IgG (ThermoFisher A11012) secondary antibody was added on cells for 1 h at room temperature (1:500 in a solution of 3% BSA, 0.05% Tween

20 in 1X PBS). Cells were washed 3 times then dried. ProLong Gold antifade reagent with DAPI (ThermoFisher P36935) was added and the microwells were sealed with coverslips. Images were captured with a confocal microscope (LSM 700, Zeiss (Toronto, ON, Canada)) and its accompanying software (ZEN, Zeiss).

Immunoprecipitation. Cell pellets were lysed with IP buffer (20 mM Tris pH 7.5, 150 mM NaCl, 2 mM EDTA, 1% NP40) containing a mixture of protease inhibitors and N-Ethyl-Maleimide (NEM, 20 mM). Proteins (500 µg) were incubated overnight with an antibody (2 µg) against SUMO2/3 (Cedarlane M114-3) or with a mouse IgG isotype control (Cedarlane 015-000-003). Complexes were incubated with protein G Dynabeads for 90 min, then washed twice with PBS (supplemented with 20 mM NEM) and once with PBS containing 0.1% Tween (supplemented with 20 mM NEM). Co-immunoprecipitated proteins were eluted from the beads with a solution of 0.2 M acidic glycine (pH 2.5). Proteins were denatured with Laemmli buffer and resolved by SDS-PAGE (7% acrylamide).

SUPPLEMENTARY REFERENCES

1. Bolger AM, Lohse M, Usadel B. Trimmomatic: a flexible trimmer for Illumina sequence data. Bioinformatics. 2014;30(15):2114-20.

2. Li H, Durbin R. Fast and accurate long-read alignment with Burrows-Wheeler transform. Bioinformatics. 2010;26(5):589-95.

3. Ramirez F, Ryan DP, Gruning B, Bhardwaj V, Kilpert F, Richter AS, et al. deepTools2: a next generation web server for deep-sequencing data analysis. Nucleic Acids Res. 2016;44(W1):W160-5.

4. Huber W, Carey VJ, Gentleman R, Anders S, Carlson M, Carvalho BS, et al. Orchestrating high-throughput genomic analysis with Bioconductor. Nat Methods. 2015;12(2):115-21.

5. Ross-Innes CS, Stark R, Teschendorff AE, Holmes KA, Ali HR, Dunning MJ, et al. Differential oestrogen receptor binding is associated with clinical outcome in breast cancer. Nature. 2012; 481(738):289-93.

6. Robinson MD, McCarthy DJ, Smyth GK. edgeR: a Bioconductor package for differential expression analysis of digital gene expression data. Bioinformatics. 2010;26(1), 139-140.

7. McCarthy, J. D, Chen, Yunshun, Smyth, K. G. Differential expression analysis of multifactor RNA-Seq experiments with respect to biological variation. Nucleic Acids Research. 2012;40(10), 4288-4297.

SUPPLEMENTARY FIGURE LEGENDS

Supplementary Figure 1. Differential effects of 4-hydroxytamoxifen and ICI182,780 on MCF-7 cell proliferation.

MCF-7 cells were seeded in estrogen-depleted media and treated with estradiol (E2, 5 nM), 4-hydroxytamoxifen (OHT, 100 nM) or ICI182,80 (ICI, 100 nM). Proliferation was assessed every 2 days by an alamarBlue® cell viability assay. A representative experiment is shown (N=3). The means ± error of 7 technical replicates are represented, along with significance (one-tailed T test, ICI vs. OHT, 8 days).

Supplementary Figure 2. ICI182,780 induces transient ERα binding to EREs in estrogen target genes in MCF-7 cells.

A. ERα binding to the *CTSD* ERE was determined by chromatin immunoprecipitation (ChIP) in MCF-7 cells treated with estradiol (E2, 5 nM) or ICI182,780 (ICI, 100 nM) for the indicated time points. Position of the *CTSD* ERE relative to the gene TSS is shown. The graph on the left shows the evolution of ERα binding over four hours after addition of E2 (green), ICI (magenta), or vehicle only (blue), while the bar graph on the right compares ERα binding in the presence of ICI to that in presence of the vehicle only. Data points from 3 independent experiments, as well as means ± SEM, are represented, along with asterisks denoting significance (one-tailed T test, ICI vs. 0): * P-value <0.05 ; ** P-value <0.005 ; *** P-value <0.0005. **B.** ERα binding to regions devoid of high-affinity EREs randomly chosen in the gene bodies of ER target genes *GREB1* and *CTSD* was evaluated by ChIP-qPCR. Data points from 3 independent experiments, as well as means ± SEM, are represented.

Supplementary Figure 3. Binding analysis of the ERα ChIP-Seq data.

ChIP-Seq was performed with an antibody against ERα on MCF-7 cells treated with estradiol (E2, 5 nM) or ICI182,780 (ICI, 100 nM) for the indicated time points (minutes) (N=3). **A-B.** Principal component analysis plot for peaks called by MACS using TMM log2-normalized read counts after subtraction of control (input DNA) read counts **A.** at 30 min and **B.** at 180 min. **C.** The location of ERα peaks called by MACS was classified as follows: gene (exon or intron), proximal (within 2 kb upstream of TSS), distal (between 10 kb upstream and 2 kb upstream of TSS), 5d (between 100 kb upstream and 10 kb upstream of TSS), gene desert (≥ 100 kb upstream or downstream of TSS), other (anything not included in the above categories). **D.** Heatmap of ChIP-Seq binding for ERα after treatment with E2, ICI182,780 or vehicle at 30 min (30') or 180 min (180'). Mapped reads in +/- 3 kb windows around ERα peaks present with E2 and ICI at 30' and containing EREs (3 260 peaks) were counted using 50 bp windows and normalized to 1x sequencing depth using deeptools2 (see Supplementary Material and Methods).

Supplementary Figure 4. Nuclear localization of ERα is not affected by treatment of MCF-7 cells by ICI182,780.

Immunofluorescence of MCF-7 cells treated with ICI (100 nM) or vehicle only (0) for the indicated time points (minutes) shows localization of endogenous ERα (red) inside the nucleus, visualized with DAPI dye (blue), for both vehicle and ICI treatment at all time points. Images are representative of 3 independent experiments.

Supplementary Figure 5. Pure AEs induce SUMOylation of ERα and accumulation of SUMO marks coinciding with induced binding of ERα at EREs in MCF-7 cells.

A. Interaction between endogenous ERα and SUMO2/3 in MCF-7 cells was assessed by co-immunoprecipitation experiments. Cells were pre-treated with the proteasome inhibitor MG132 (10 µM) for 2 h and then treated with ICI182,780 (ICI, 100 nM) or vehicle only (0) for 30 min. ERα levels in input samples, samples immunoprecipitated with a SUMO2/3 antibody or with control IgG were assessed by Western analysis. Two different film exposures are shown to reveal both ERα and its modified forms. A representative experiment is shown (N=2). **B.** Transcript abundance (TPM) of the SUMO paralogs in MCF-7 cells, according to our transcriptome data. The means from 3 independent RNA-seq data sets ± SEM are represented. **C.** ERα and SUMO2/3 binding to the *CTSD* ERE was determined by ChIP-qPCR in MCF-7 cells treated with 100 nM of 4-hydroxytamoxifen (OHT), ICI182,780 (ICI) or RU58668 (RU58) for the indicated time points (minutes). Data points from 3 independent experiments, as well as means ± SEM, are represented. Asterisks denote significance (one-tailed T test, AE vs. 0): * P-value <0.05. **D.** SUMO2/3 binding to regions devoid of high-affinity EREs randomly selected in the gene bodies of ER target genes *GREB1* and *CTSD* was determined by ChIP-qPCR. Data points from 2 independent experiments, as well as means ± SEM, are represented. **E.** ERα (N=3) or SUMO2/3 (N=2) binding to EREs of E2 target genes *CA12*, *CDH26*, *FOXA1* and *ITGB6* was determined by ChIP-qPCR in MCF-7 cells treated with ICI182,780 (ICI, 100 nM) for the indicated time points. Data points from independent experiments, as well as means ± SEM, are represented. Asterisks denote significance (one-tailed T test, ICI vs. 0): * P-value <0.05 ; ** P-value <0.005.

Supplementary Figure 6. ICI182,780 treatment of MCF-7 cells alters SUMO2/3 distribution on chromatin.

MCF-7 cells were treated with ICI182,780 (ICI, 100 nM) for the indicated time points (minutes). ChIP-Seq SUMO2/3 was performed (N=2). **A.** Principal component analysis plot for peaks called by MACS using TMM log2-normalized read counts after subtraction of control (input DNA) read counts **B.** Location of SUMO2/3 peaks called by MACS was classified as follows: gene (exon or intron), proximal (within 2 kb upstream of TSS), distal (between 10 kb upstream and 2 kb upstream of TSS), 5d (between 100 kb upstream and 10 kb upstream of TSS), gene desert (≥ 100 kb upstream or downstream of TSS), other (anything not included in the above categories). **C.** Heatmap of ChIP-Seq binding for SUMO2/3 after treatment with ICI182,780 or vehicle at 30 min (30') or 180 min (180'), as indicated. Mapped reads in +/- 3 kb windows around the top 5 % of ERα peaks present with E2 and ICI at 30' (583 peaks) were counted and normalized to 1x sequencing depth using deeptools2 (see Supplementary Material and Methods). **D.** UCSC browser snapshots of ERα and SUMO2/3 peaks at EREs near E2 target genes *CTSD* and *CDH26* for the vehicle and ICI conditions.

Supplementary Figure 7. Chromatin at ER target regions is less accessible in MCF-7 cells following ICI182,780 treatment.

A. Levels of accessible chromatin at the *CTSD* ERE were assessed by Formaldehyde-Assisted Isolation of Regulatory Elements (FAIRE) in MCF-7 cells treated with estradiol (E2, 5 nM), 4-hydroxytamoxifen (OHT, 100 nM), ICI182,780 (ICI, 100 nM) or vehicle only (0) for the indicated time points (20 or 60 min). Data points from 3 independent experiments, as well as

means ± SEM, are represented. **B.** Levels of accessible chromatin at the *CTSD* ERE were assessed by FAIRE in MCF-7 Tet-ON SENP1-FLAG cells induced with doxycycline (DOX, 3 μg/mL) or not (0) for 24 h, and subsequently treated with E2 (5 nM), OHT (100 nM), ICI (100 nM) or vehicle only (0) for 1 h. Data points from 3 independent experiments, as well as means ± SEM, are represented. Asterisks denote significance (one-tailed T test, vs. 0): * P-value <0.05 ; ** P-value <0.005. **C.** Levels of accessible chromatin at regions devoid of high-affinity EREs randomly selected in the gene bodies of ER target genes *GREB1* and *CTSD* were assessed by FAIRE in MCF-7 cells treated as in (**A**). Data points from 3 independent experiments, as well as means ± SEM, are represented. **D.** Levels of accessible chromatin at various EREs of E2 target genes was assessed by FAIRE in MCF-7 cells treated as in (**A**). Data points from 3 independent experiments, as well as means ± SEM, are represented. Asterisks denote significance (one-tailed T test, vs. 0): * P-value <0.05.

Supplementary Figure 8. ICI182,780 induces transient binding of ERα to DNA and leads to chromatin closure at EREs in MCF-7 cells grown in complete media.

A. ERα binding to EREs upstream of the *TFF1* and *GREB1* genes was determined by ChIP-qPCR in MCF-7 cells cultured in complete media without estrogenic depletion and treated with ICI182,780 (ICI, 100 nM) or with vehicle only (0) for the indicated time points (min). Data points from 3 independent experiments, as well as means ± SEM, are represented. Asterisks denote significance (one-tailed T test, ICI vs. 0): * P-value <0.05 ; *** P-value <0.0005. **B.** Levels of accessible chromatin at ER target regions was assessed by FAIRE in MCF-7 cells cultured in complete media and treated with estradiol (E2, 5 nM), ICI182,780 (ICI, 100 nM) or

vehicle only (0) for 3 h. Data points from 2 independent experiments, as well as means ± SEM, are represented. Asterisks denote significance (one-tailed T test, vs. 0): * P-value <0.05.

Supplementary Figure 9. ERα(V534E) is not SUMOylated in the presence of ICI182,780. Interaction between ERα and SUMO3 was assessed by BRET[1] assays (N=2) in HEK-293 cells co-transfected with wt or mutant ERα-*R*lucII and YFP-SUMO3 and treated with ICI182,780 (ICI, 1 μM) or vehicle only (0) for the indicated time points.

Supplementary Figure 10. Uncropped scans of Western Blot films.
