# Supplementary material for: Role of SUMOylation in differential ERα transcriptional repression by tamoxifen and fulvestrant in breast cancer cells
Source: Oncogene. 2018 Sep 6;38(7):1019–37. doi: 10.1038/s41388-018-0468-9 (PMC6514857; doi:10.1038/s41388-018-0468-9)
Supplement: Supplementary file 2 — Supplementary figures [file 41388_2018_468_MOESM2_ESM.pdf]

Supplementary Figure 1.

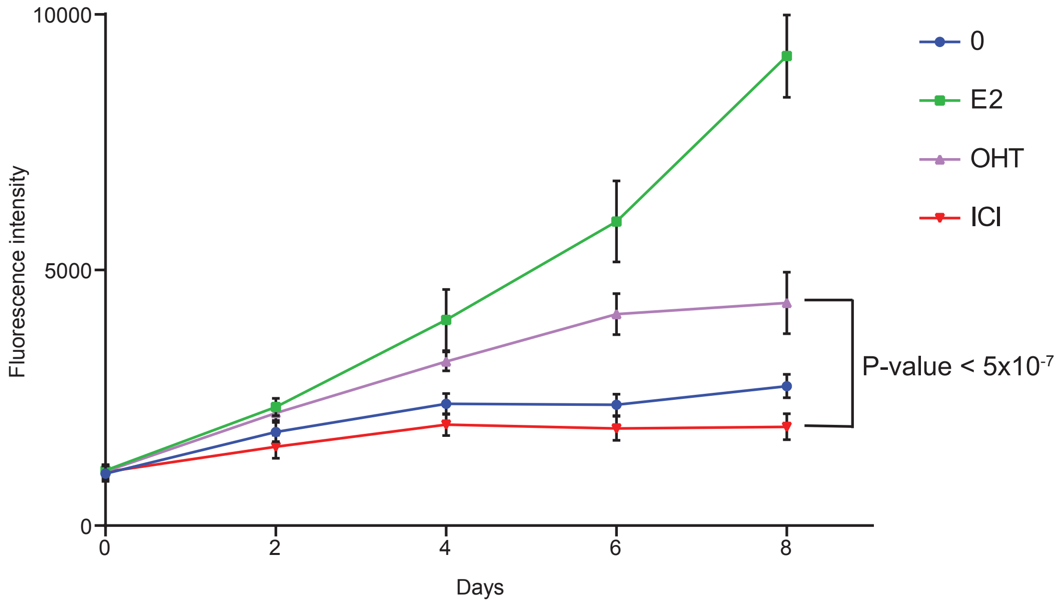

Supplementary Figure 2.

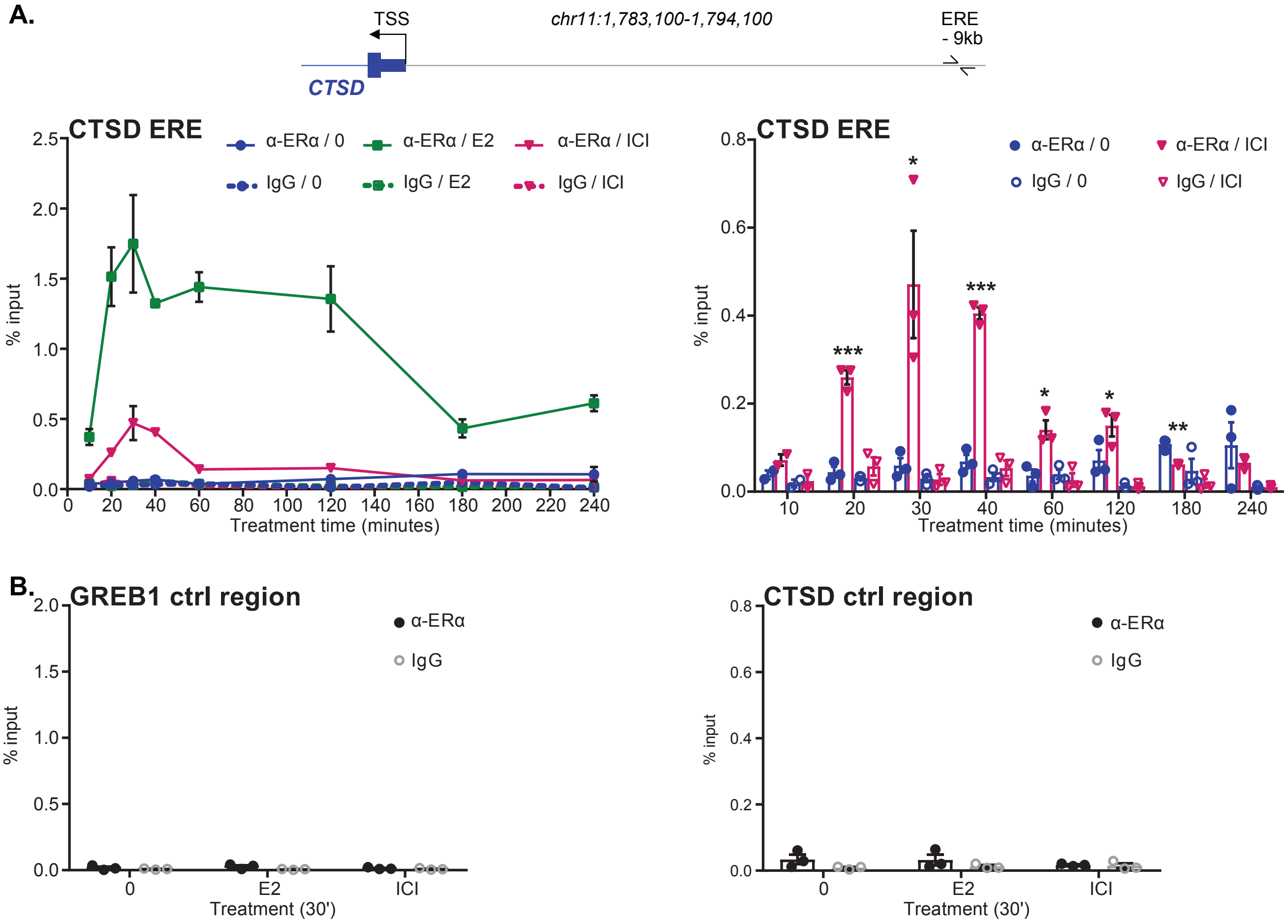

Supplementary Figure 3.

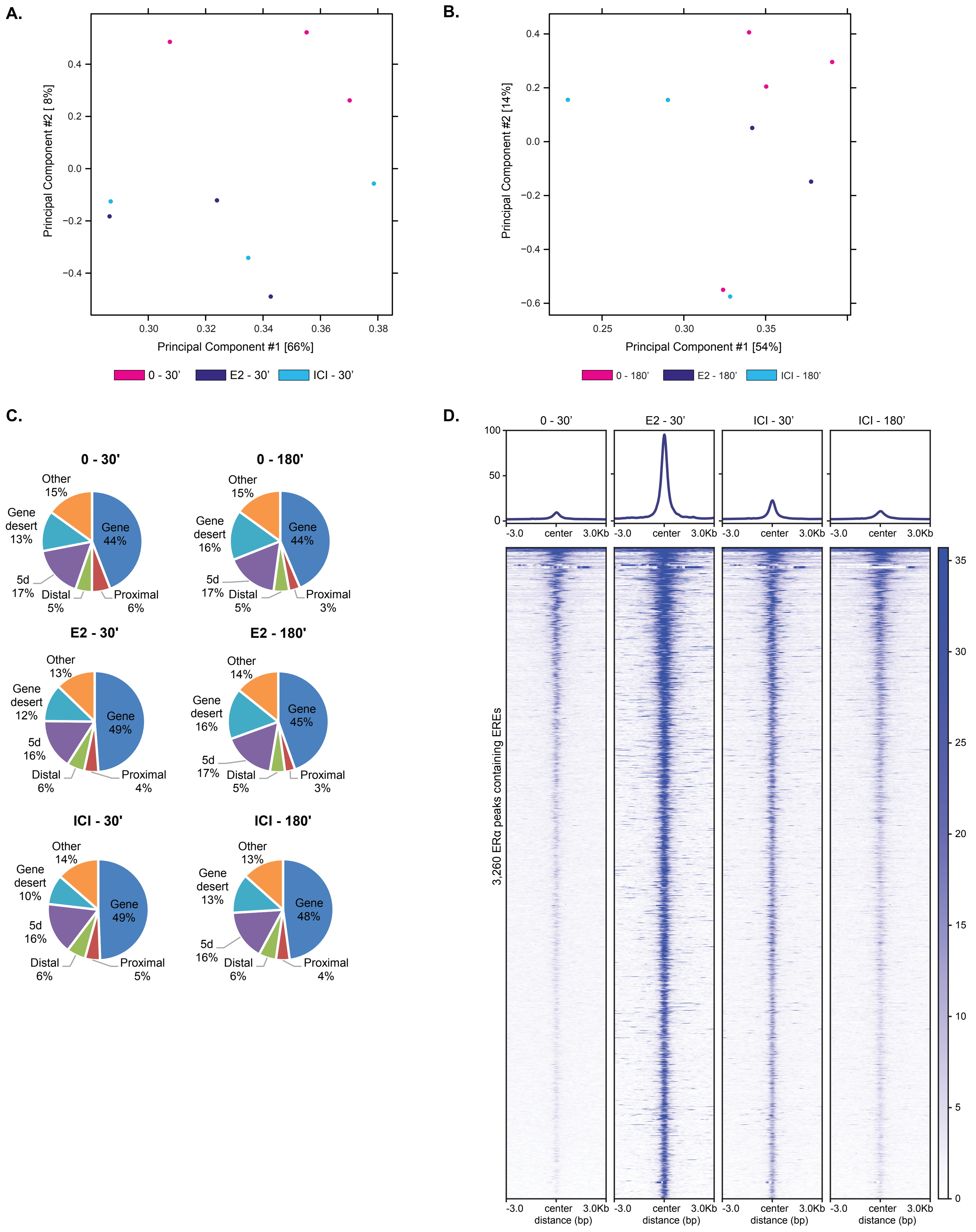

Supplementary Figure 4.

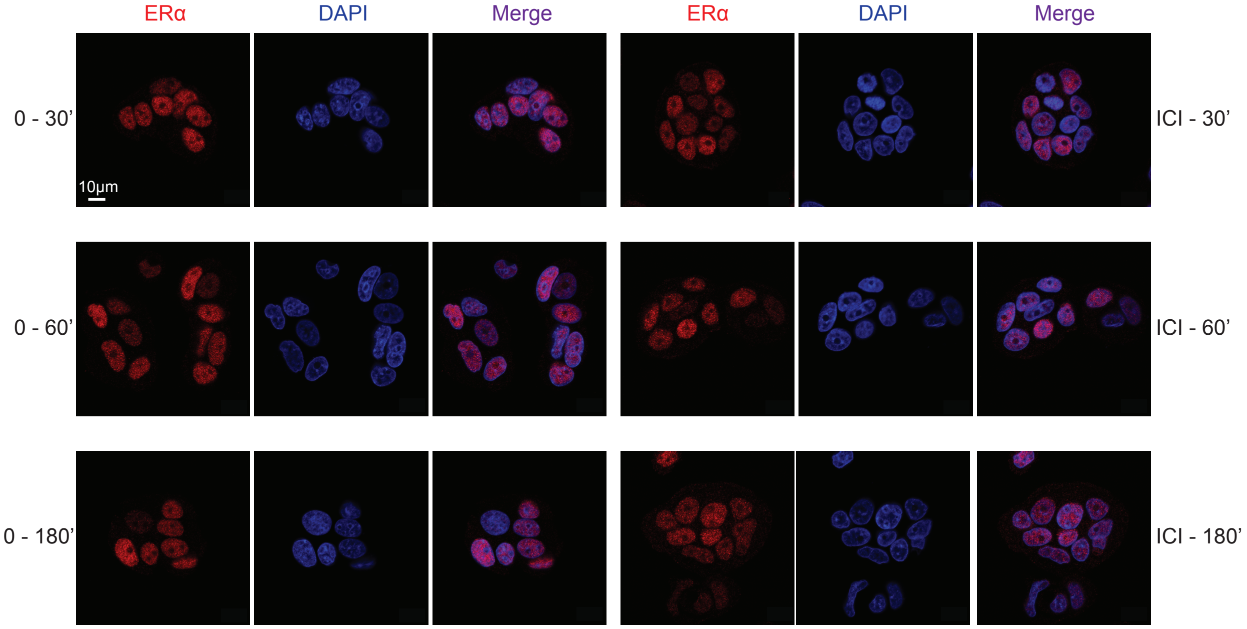

Supplementary Figure 5.

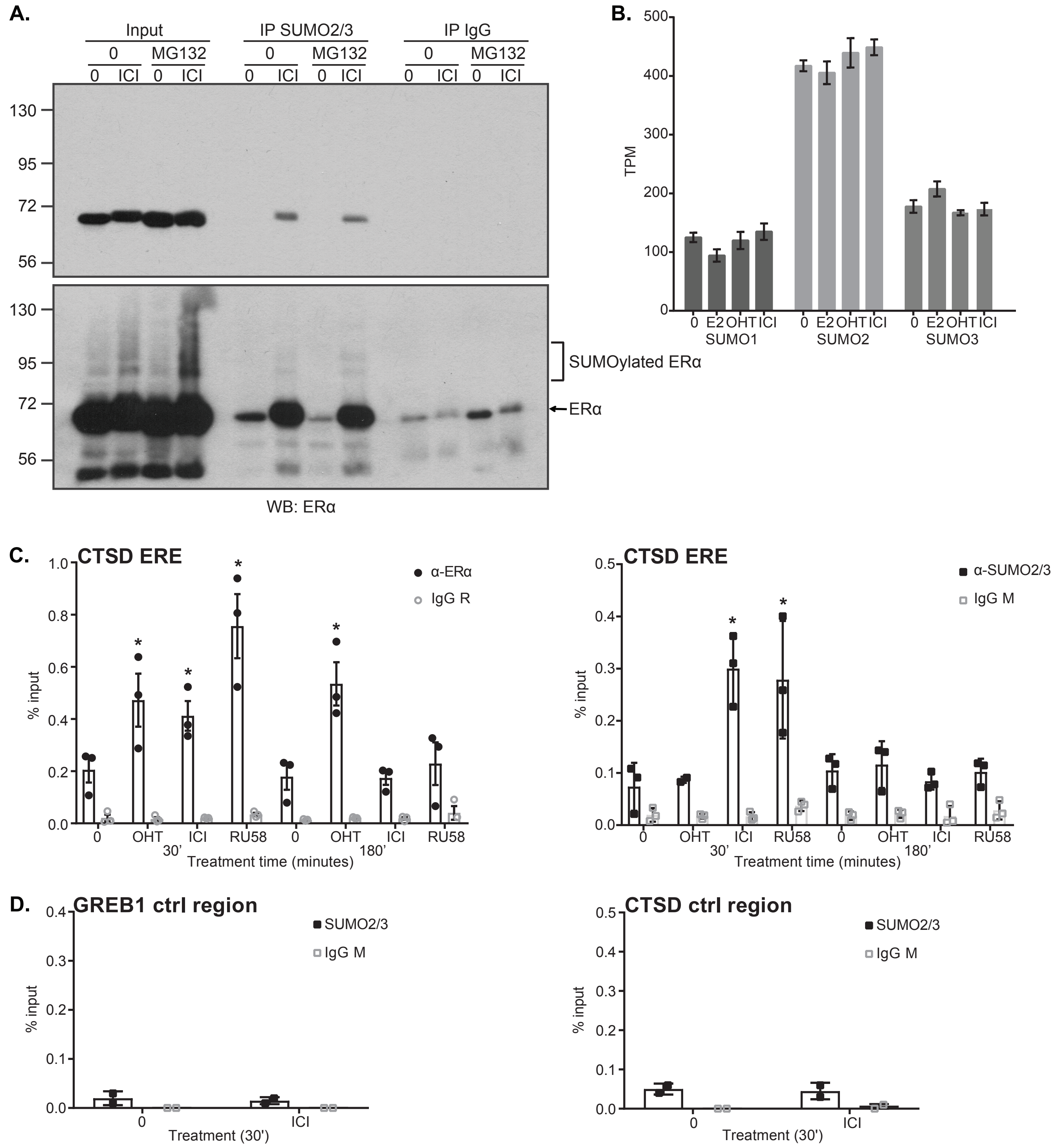

E.

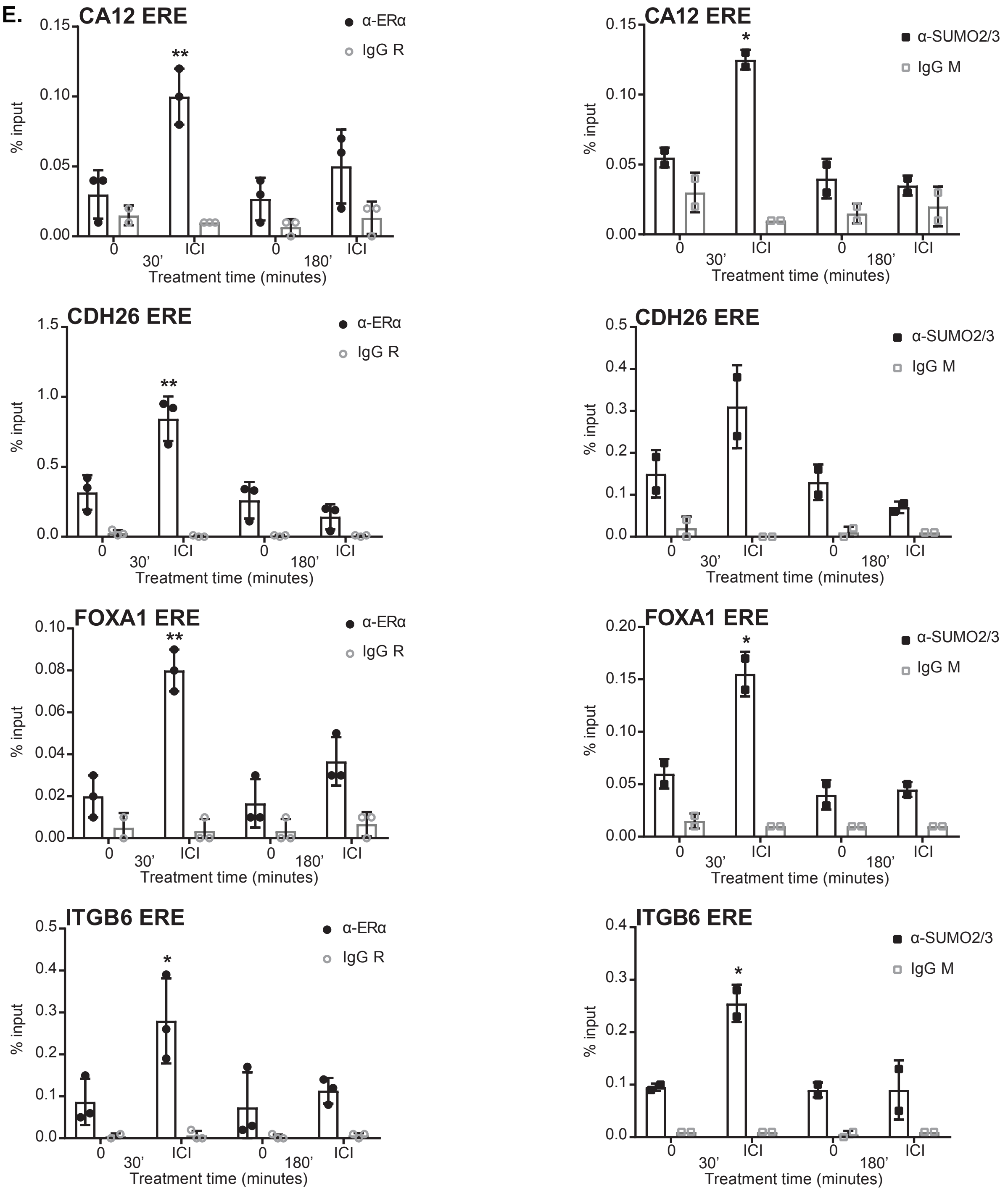
CA12 ERE
α-ERα
IgG R
**
% input
0.15
0.10
0.05
0.00
0
30’
ICI
0
180’
ICI
Treatment time (minutes)
CA12 ERE
α-SUMO2/3
IgG M
*
% input
CDH26 ERE
α-ERα
IgG R
**
1.5
1.0
0.5
0.0
CDH26 ERE
α-SUMO2/3
IgG M
0.5
0.4
0.3
0.2
0.1
0.0
FOXA1 ERE
α-ERα
IgG R
**
0.10
0.08
0.06
0.04
0.02
0.00
FOXA1 ERE
α-SUMO2/3
IgG M
*
0.20
0.15
0.10
0.05
0.00
ITGB6 ERE
α-ERα
IgG R
*
0.5
0.4
0.3
0.2
0.1
0.0
ITGB6 ERE
α-SUMO2/3
IgG M
*
0.4
0.3
0.2
0.1
0.0

Supplementary Figure 6.

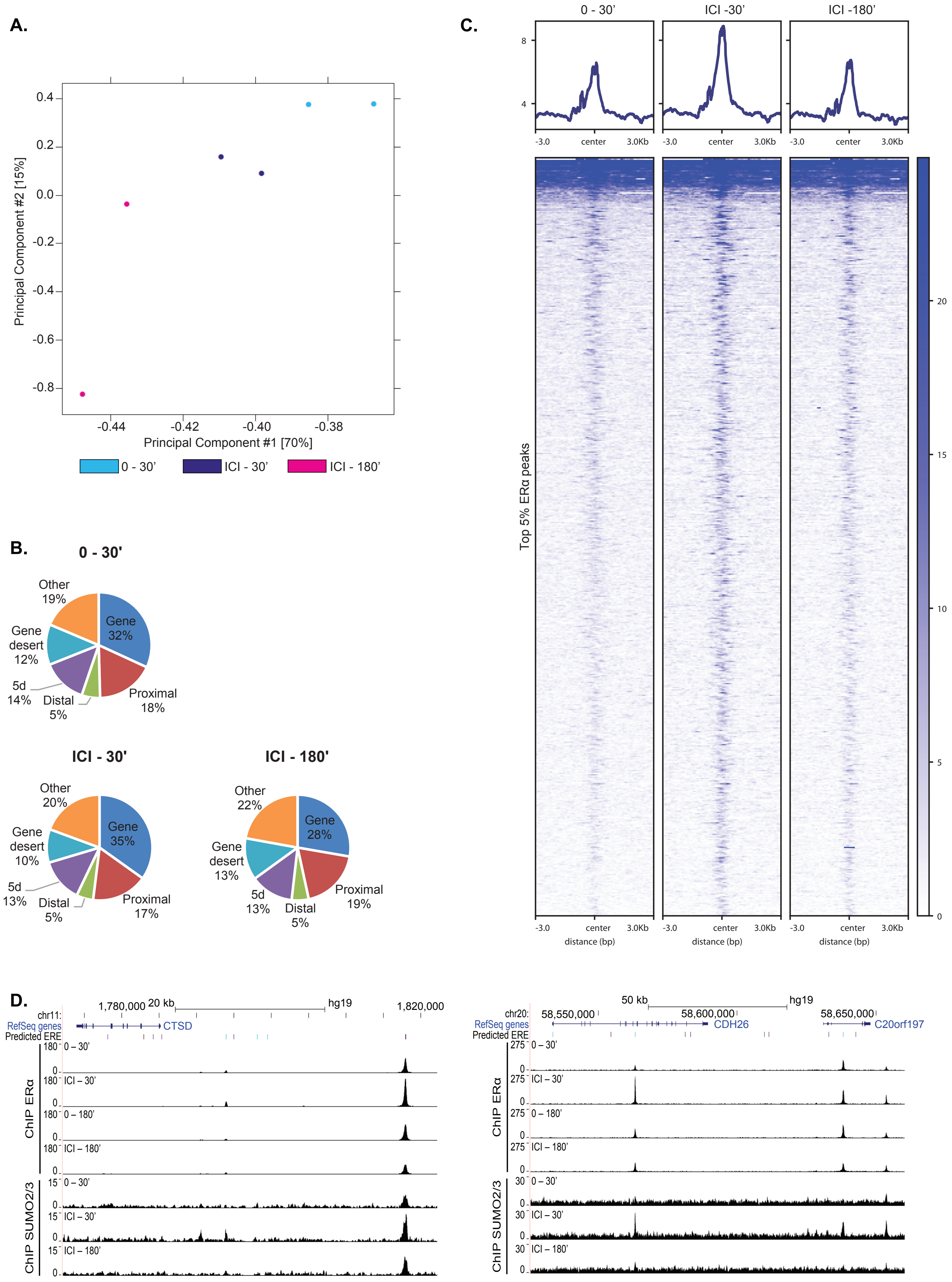

Supplementary Figure 7.

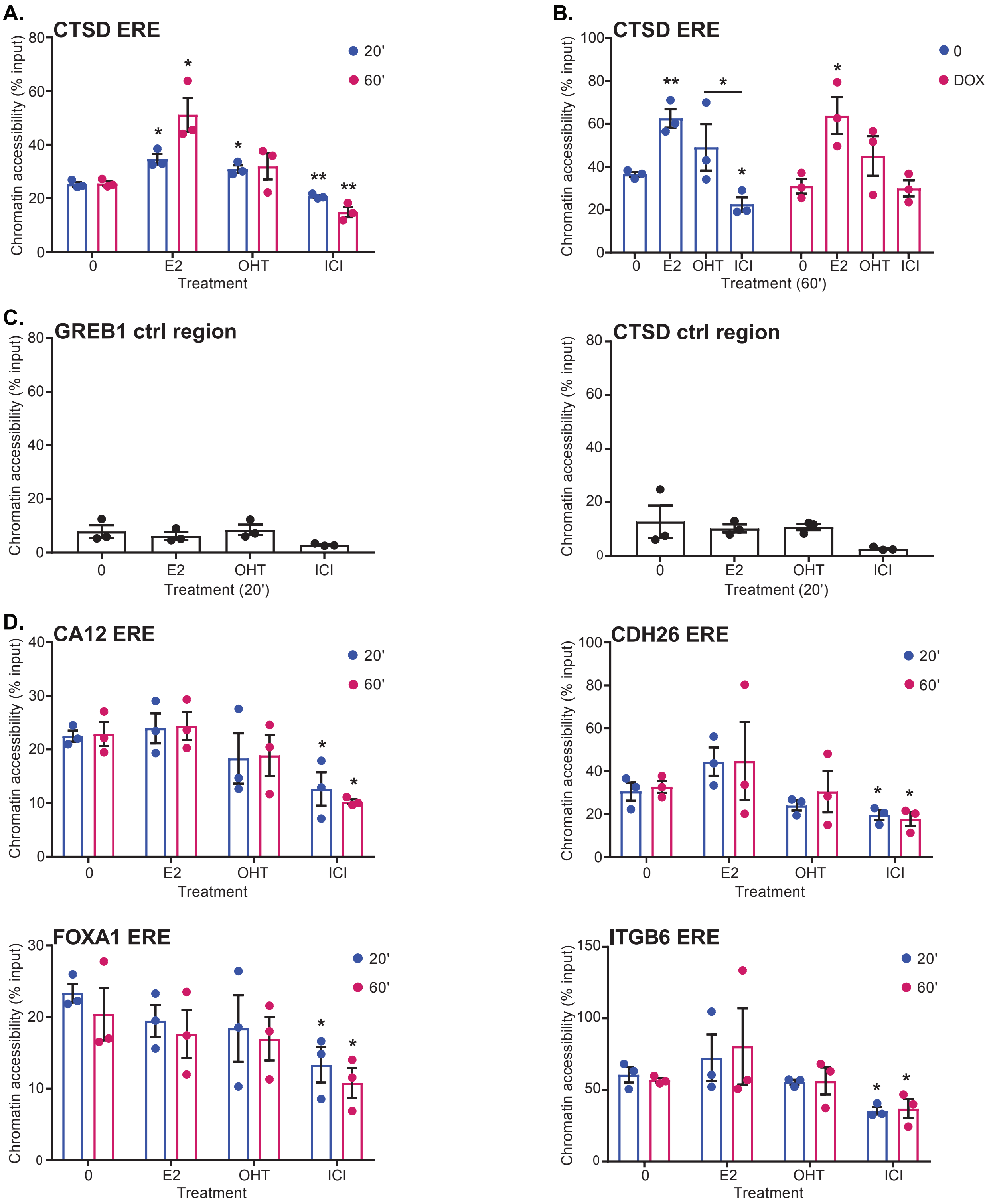

Supplementary Figure 8.

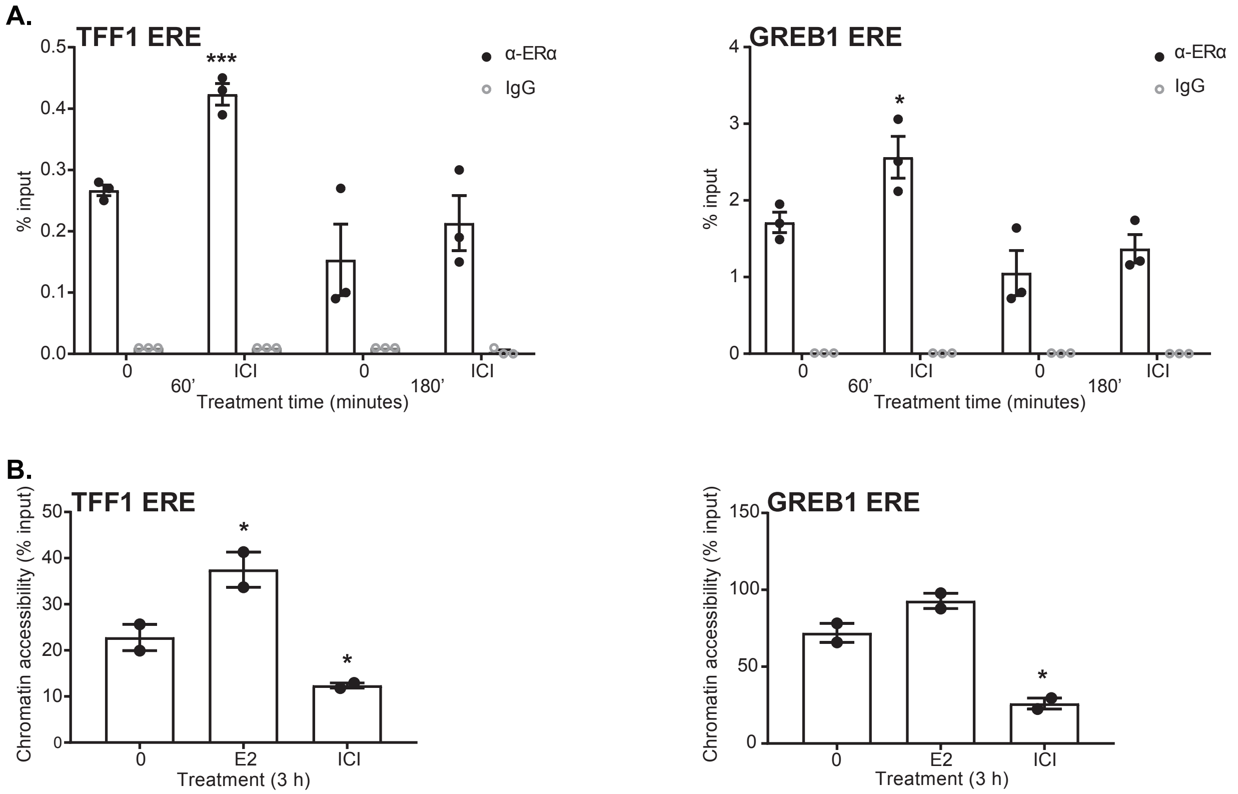

Supplementary Figure 9.

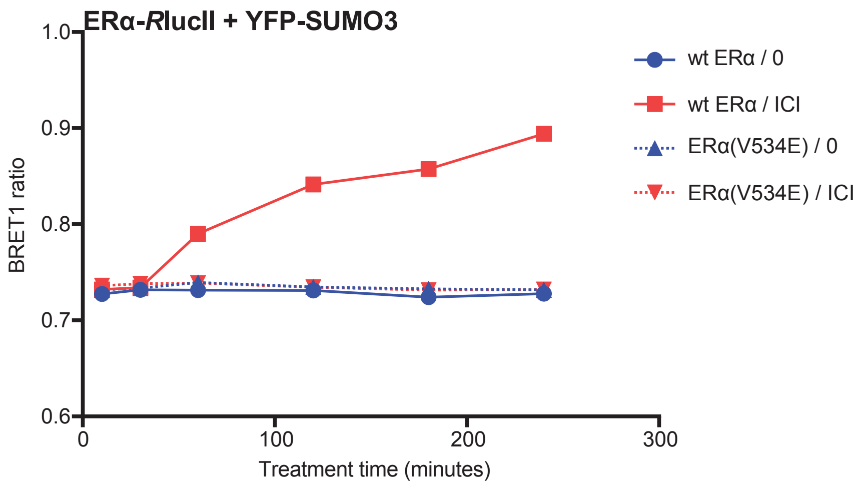

Fig. 4A

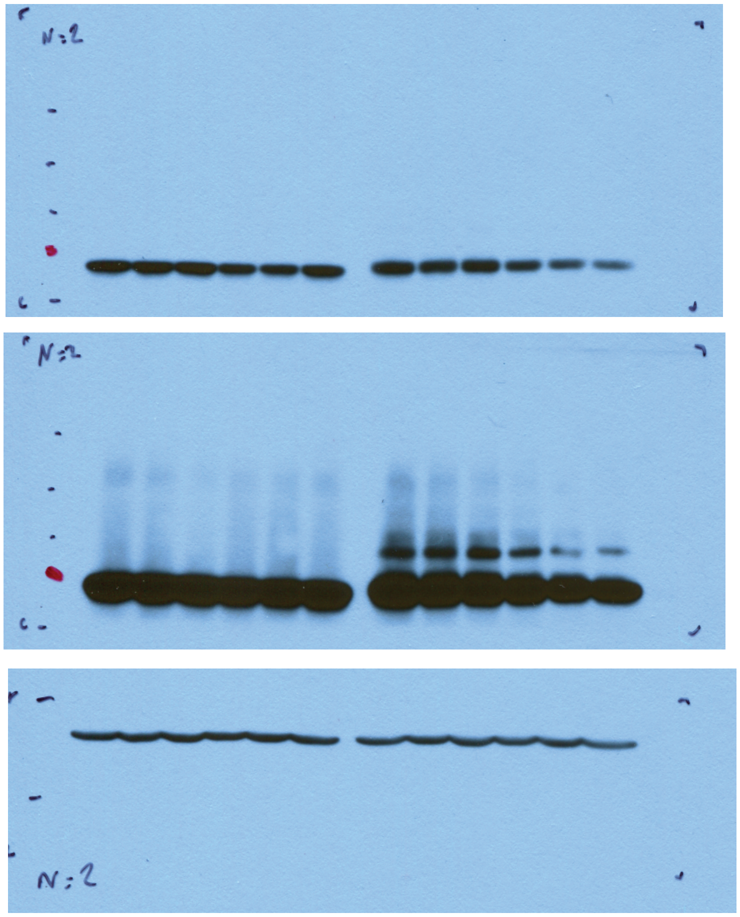

Fig. 4B

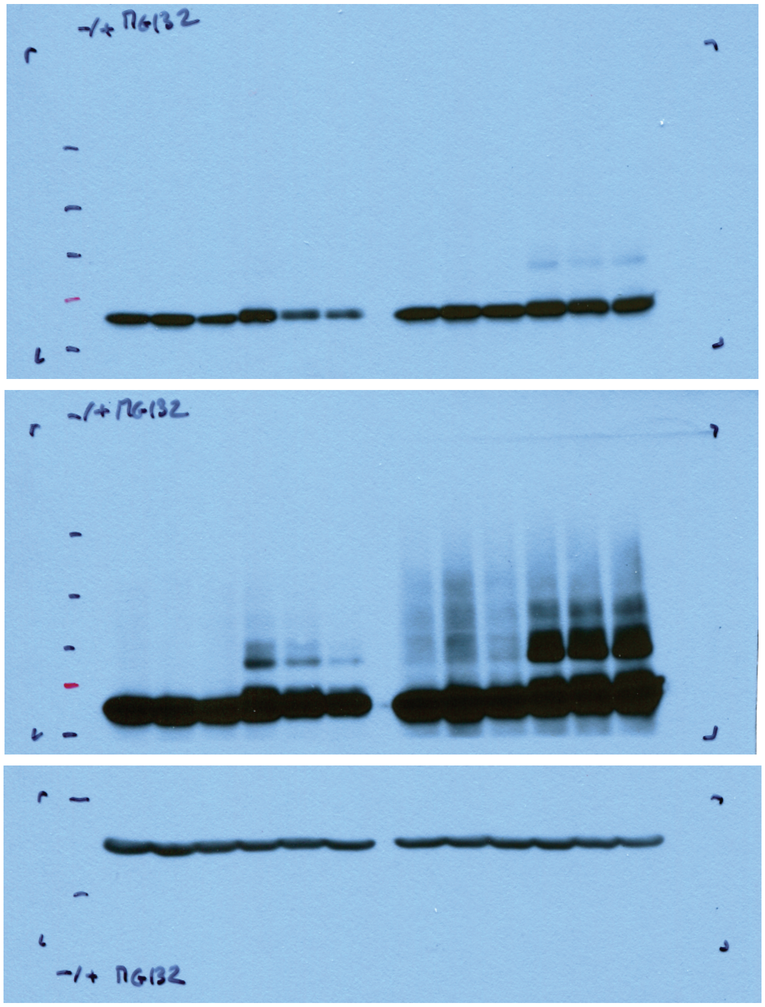

Fig. 5B

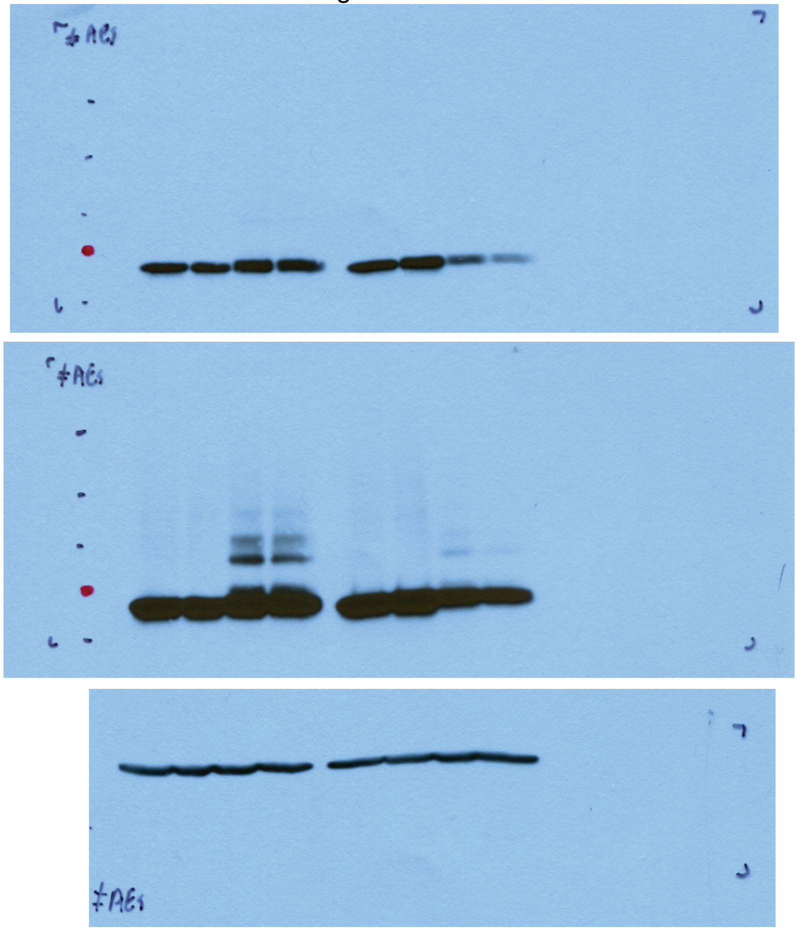

Fig. 5C

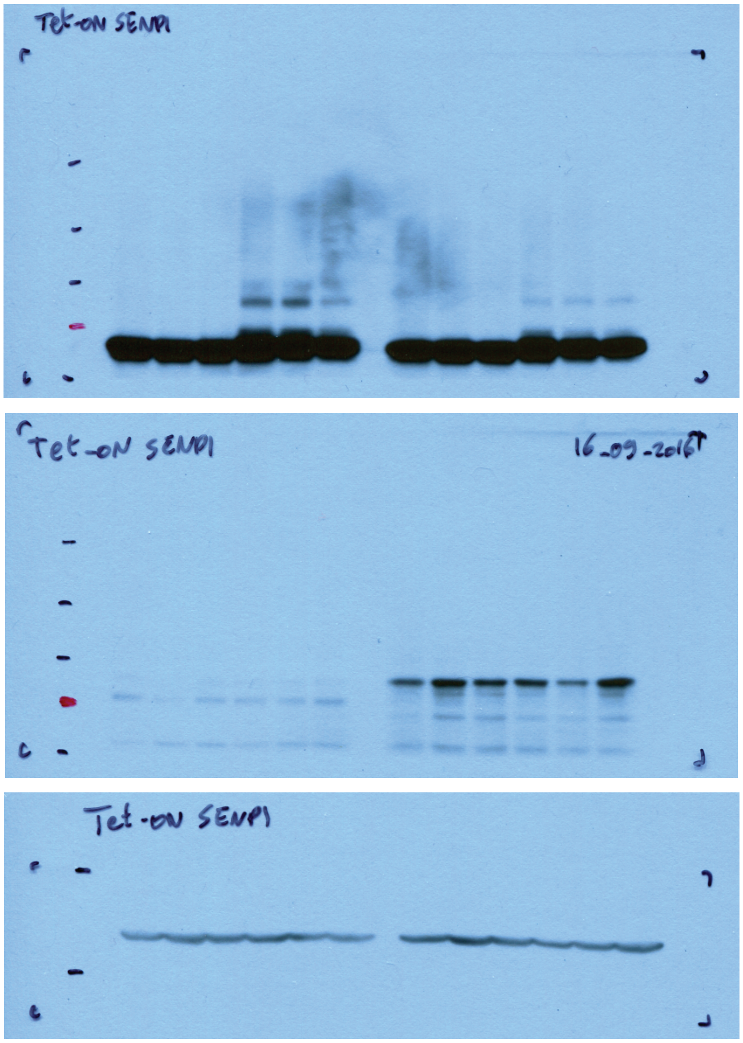

Fig. 8B

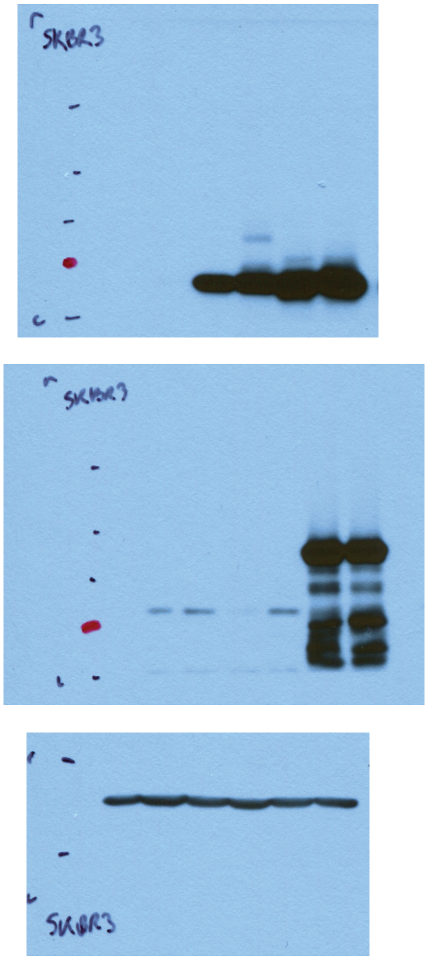

Fig. 9B

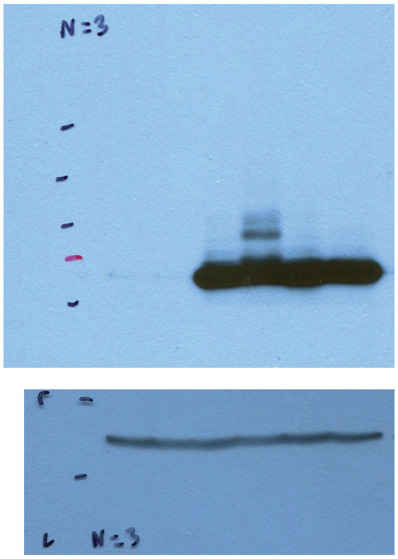

Suppl. Fig. 5A

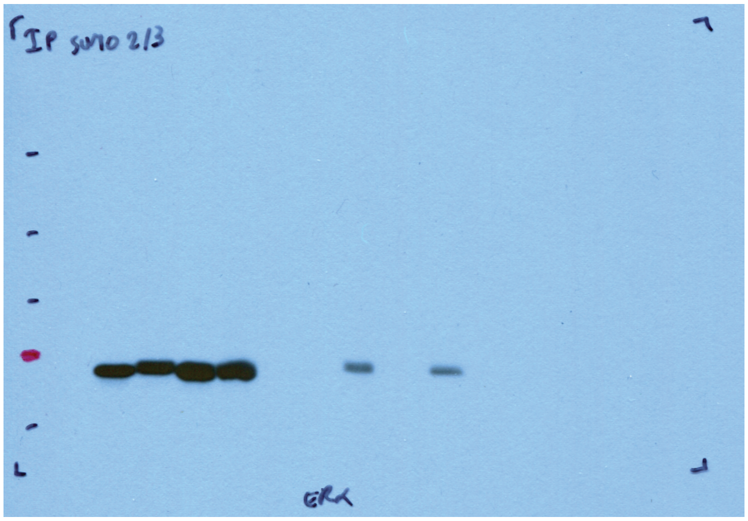

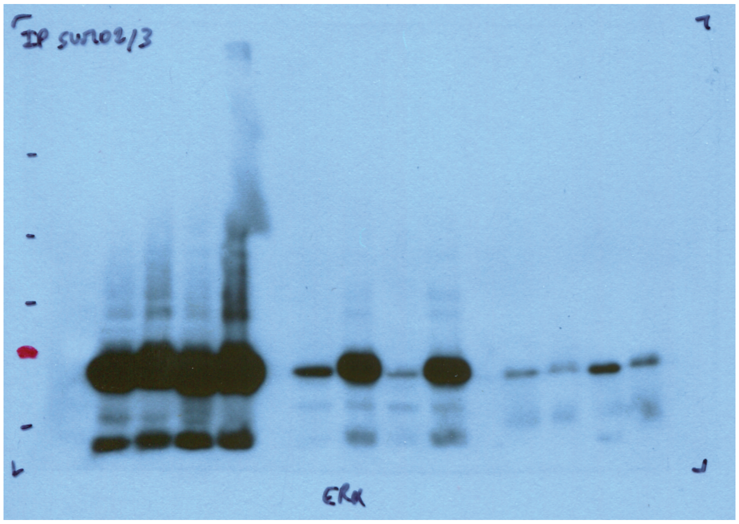

Supplementary Figure 10.
