# Supplementary material for: Role of SUMOylation in differential ERα transcriptional repression by tamoxifen and fulvestrant in breast cancer cells
Source: Oncogene. 2018 Sep 6;38(7):1019–37. doi: 10.1038/s41388-018-0468-9 (PMC6514857; doi:10.1038/s41388-018-0468-9)
Supplement: Supplementary file 4 — Supplementary Tables 2–5 [file 41388_2018_468_MOESM4_ESM.pdf]

Supplementary Table 2. ChIP-Seq alignment and duplication metrics.

ChIP	Treatment condition	GEO sample name	Number of raw reads	Aligned filtered reads %	Duplication %	Number of called peaks (MACS)
ChIP ERα	0 (30’)	DSP250_EtOH_ERa	72 327 936	93.2	26.6	3 081
		DSP488_V_30_ERa_N1	79 044 004	74.4	5.0	
		DSP514_V_30_ERa_N1	75 179 476	79.5	6.4	
	E2 (30’)	DSP250_E2_ERa	74 486 428	93.5	58.3	32 188
		DSP460_E2_30_ERa_N2	74 935 856	69.3	75.9	
		DSP488_E2_30_ERa_N1	88 837 892	78.2	8.0	
	ICI (30’)	DSP250_ICI_ERa	73 358 972	92.9	37.9	11 965
		DSP488_ICI_30_ERa_N2	66 190 498	63.0	5.7	
		DSP514_ICI_30_ERa_N1	88 510 816	78.6	3.8	
	0 (180’)	DSP488_V_3h_ERa_N1	66 387 928	73.2	69.1	7 416
		DSP488_V_3h_ERa_N2	66 399 142	81.2	9.5	
		DSP514_V_3h_ERa_N1	80 065 126	60.3	4.8	
		DSP514_V_3h_ERa_N2	75 134 710	75.1	4.5	
	E2 (180’)	DSP488_E2_3h_ERa_N1	78 514 428	66.9	11.7	11 777
		DSP488_E2_3h_ERa_N2	62 754 712	80.0	14.8	
	ICI (180’)	DSP488_ICI_3h_ERa_N1	93 340 098	77.3	6.6	7 769
		DSP514_ICI_3h_ERa_N1	80 122 622	41.5	5.6	
		DSP514_ICI_3h_ERa_N2	51 775 844	77.9	23.6	
ChIP SUMO2/3	0 (30’)	DSP514_V_30_SUMO_N1	86 070 076	81.3	2.8	3 014
		DSP514_V_30_SUMO_N2	84 033 204	84.0	3.1	
	ICI (30’)	DSP514_ICI_30_SUMO_N1	71 253 026	86.4	2.3	4 507
		DSP514_ICI_30_SUMO_N2	75 284 796	84.3	3.0	
	ICI (180’)	DSP514_ICI_3h_SUMO_N1	87 030 066	81.2	2.8	1 129
		DSP514_ICI_3h_SUMO_N2	69 469 102	82.2	2.8	
	input	DSP250_input	46 928 800	94.6	5.9	
		DSP460_input	69 560 840	91.8	27.7	
		DSP488_input	52 631 920	92.2	1.8	
		DSP514_input	69 733 660	92.3	1.7	

Number of raw reads: Total number of reads obtained from the sequencer
Aligned filtered reads %: (Number of aligned reads to the reference after filtering by mapping quality) / (Number of remaining reads after the trimming step)
Duplication %: (Number of aligned reads having the same 5' alignment positions (for both mates in the case of paired-end reads) after filtering by mapping quality) / (Number of aligned filtered reads)

Supplementary Table 3. Motif enrichment in SUMO2/3 peaks.

0 (30')	ICI (30')	In ERα peaks	ICI (180')
**CTCF element** 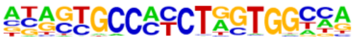 10^{-459}	**CTCF element** 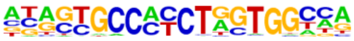 10^{-602}	/	**CTCF element** 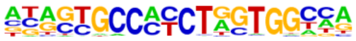 10^{-159}
**BORIS / CTCFL element** 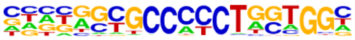 10^{-363}	**BORIS / CTCFL element** 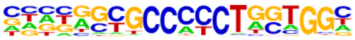 10^{-446}	/	**BORIS / CTCFL element** 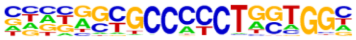 10^{-125}
**STAT6 element** 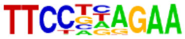 10^{-29}	Fosl2 element 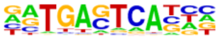 10^{-59}	10^{-355}	
**TEAD4 element** 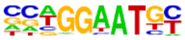 10^{-28}	Fra1 element 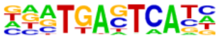 10^{-56}	10^{-335}	
**GATA1 element** 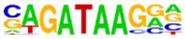 10^{-27}	Jun-AP1 element 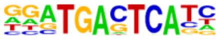 10^{-55}	10^{-325}	
GATA2 element 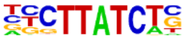 10^{-23}	BATF element 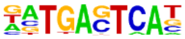 10^{-55}	10^{-335}	
**TEAD element** 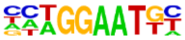 10^{-22}	Atf3 element 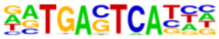 10^{-47}	10^{-322}	
STAT5 element 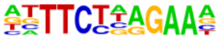 10^{-20}	AP-1 element 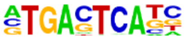 10^{-43}	10^{-315}	
	Bach2 element 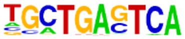 10^{-32}	10^{-184}	
	Estrogen Response element 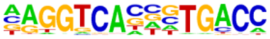 10^{-32}	10^{-1359}	
	**TEAD4 element** 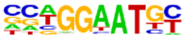 10^{-27}	10^{-141}	
	**STAT6 element** 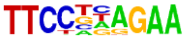 10^{-23}	/	
	**TEAD element** 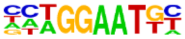 10^{-22}	10^{-113}	
	Foxa2 element 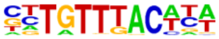 10^{-22}	10^{-494}	
	**Gata1 element** 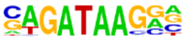 10^{-21}	10^{-134}	
	AP-2 element 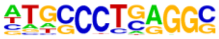 10^{-21}	10^{-231}	

Motifs enriched in the different SUMO2/3 ChIP-Seq data sets (p-value < 10^{-20}) are indicated.
Motifs in common between the SUMO2/3 data sets under different treatment conditions are bolded.
Motifs in common between the SUMO2/3 and the ERα ChIP-Seq data sets for ICI (30') are indicated along with their p-value.

Supplementary Table 4. Primers used for RT-qPCR.

Studied region	Primers		UPL probe (Roche)
mRNA *RPLP0*	Forward	TCCCACTTGCTGAAAAGGTC	#74
	Reverse	AGCAGGAGCAGCTGTGGT	
mRNA *TBP*	Forward	GAACATCATGGATCAGAACAACA	#87
	Reverse	ATAGGGATTCCGGGAGTCAT	
mRNA *GREB1*	Forward	CCACAAAGGGTGGTCTCCAGAA	#77
	Reverse	CACTGGCTTGGCCTTGCATATT	
mRNA *XBP1*	Forward	CCCTGGTTGCTGAAGAGG	#62
	Reverse	TGGAGGGGTGACAACTGG	
mRNA *CTSD*	Forward	GCCTACTGGCAGGTCCAC	#10
	Reverse	GTGTCCACAATGGCCTCAC	
mRNA *AGR3*	Forward	AGGCTCATATGTGTACAATCTGTTAGA	#11
	Reverse	TGGGCAATATGTGCCTAGAA	
mRNA *E2F1*	Forward	TCCAAGAACCACATCCAGTG	#5
	Reverse	CTGGGTCAACCCCTCAAG	
mRNA *MYBL2*	Forward	CCCGAGAAGCAGAAGAGGA	#26
	Reverse	GCCAGAGACTTCCGGACTTT	
mRNA *TFF1*	Forward	ACCATGGAGACAAGGTGAT	#66
	Reverse	AAATTCACACTCCTCTTCTG	

Supplementary Table 5. Primers used for ChIP-qPCR and FAIRE-qPCR.

Studied region	Primers		UPL probe (Roche)
ERE -400bp *TFF1*	Forward	TTGGCCGTGACAACAGTG	#12
	Reverse	CTAGACGGAATGGGCTTCAT	
ERE -1.5kb *GREB1*	Forward	CTGACCTAGAAGCAACCAAAATACT	#84
	Reverse	GGCAGCAAACTTGTTTAGGTATG	
ERE -9kb *CTSD*	Forward	CTCCCTCCTCTTAGGGCTGA	#49
	Reverse	AGCCCCCTTTCTCTTGAGG	
GREB1 ctrl region (non-ERE site +55kb)	Forward	ACACGAGCCGTTCCAGAAT	#19
	Reverse	CCAGGGTAGCCAAAATAGCA	
CTSD ctrl region (non-ERE site +4.5kb)	Forward	GTGCTTCACAGTCGTCTTCG	#25
	Reverse	GGTTCGTGACTCACAGCAAG	
ERE +1.2kb *CA12*	Forward	GAGGCTCAGAGCTTTTATGCTG	#77
	Reverse	GAAAGTGATTTTACTGGCTGAAACT	
ERE +30kb *CDH26*	Forward	AGATTCGCCTGCCCATTT	#24
	Reverse	CTGGTGCAGCGTCTACAGAG	
ERE -1kb *FOXA1*	Forward	TCTGCACTGCGAAAGAGATG	#29
	Reverse	GGTCACAGACAGGACCAAGC	
ERE -22kb *ITGB6*	Forward	GAATGTGGGTGAAGGGTGAG	#27
	Reverse	TGCAGGGAAGGGTTAGGTC	
